# Phase 1 Study of the E-Selectin Inhibitor GMI 1070 in Patients with Sickle Cell Anemia

**DOI:** 10.1371/journal.pone.0101301

**Published:** 2014-07-02

**Authors:** Ted Wun, Lori Styles, Laura DeCastro, Marilyn J. Telen, Frans Kuypers, Anthony Cheung, William Kramer, Henry Flanner, Seungshin Rhee, John L. Magnani, Helen Thackray

**Affiliations:** 1 Division of Hematology Oncology, University of California Davis School of Medicine, Sacramento, California, United States of America; 2 Clinical and Translational Sciences Center, UC Davis School of Medicine, Sacramento, California, United States of America; 3 Department of Pathology and Laboratory Medicine, UC Davis School of Medicine, Sacramento, California, United States of America; 4 VA Northern California Health Care System, Sacramento, California, United States of America; 5 Children's Hospital and Research Institute Oakland, Oakland, California, United States of America; 6 Division of Hematology and Oncology, University of Pittsburgh, Pittsburgh, Pennsylvania, United States of America; 7 Kramer Consulting LLC, North Potomac, Maryland, United States of America; 8 GlycoMimetics, Inc, Gaithersburg, Maryland, United States of America; 9 Rho, Inc., Chapel Hill, North Carolina, United States of America; Cardiff University, United Kingdom

## Abstract

**Background:**

Sickle cell anemia is an inherited disorder of hemoglobin that leads to a variety of acute and chronic complications. Abnormal cellular adhesion, mediated in part by selectins, has been implicated in the pathophysiology of the vaso-occlusion seen in sickle cell anemia, and selectin inhibition was able to restore blood flow in a mouse model of sickle cell disease.

**Methods:**

We performed a Phase 1 study of the selectin inhibitor GMI 1070 in patients with sickle cell anemia. Fifteen patients who were clinically stable received GMI 1070 in two infusions.

**Results:**

The drug was well tolerated without significant adverse events. There was a modest increase in total peripheral white blood cell count without clinical symptoms. Plasma concentrations were well-described by a two-compartment model with an elimination T_1/2_ of 7.7 hours and CLr of 19.6 mL/hour/kg. Computer-assisted intravital microscopy showed transient increases in red blood cell velocity in 3 of the 4 patients studied.

**Conclusions:**

GMI 1070 was safe in stable patients with sickle cell anemia, and there was suggestion of increased blood flow in a subset of patients. At some time points between 4 and 48 hours after treatment with GMI 1070, there were significant decreases in biomarkers of endothelial activation (sE-selectin, sP-selectin, sICAM), leukocyte activation (MAC-1, LFA-1, PM aggregates) and the coagulation cascade (tissue factor, thrombin-antithrombin complexes). Development of GMI 1070 for the treatment of acute vaso-occlusive crisis is ongoing.

**Trial Registration:**

ClinicalTrials.gov NCT00911495

## Introduction

Sickle cell disease (SCD) results from a mutation in the β-globin gene that leads to a substitution of valine for glutamic acid at position 6 of the β globin chain. The most common genotypes associated with disease are homozygous S (HbSS), and compound heterozygous hemoglobin SC and S/β-thalassemia.[1], [2] The prevalence in the United States is between 70,000–100,000, but is much greater in Africa and other parts of the world. Complications are protean and affect every major organ system including acute and chronic pain; ischemic and hemorrhagic stroke; infections; acute chest syndrome and pulmonary hypertension; congestive heart failure; azotemia, proteinuria, renal concentrating defects, papillary necrosis, and priapism; osteomyelitis and avascular necrosis of the bone; and leg ulcers. Vascular occlusion (or vaso-occlusive crisis – VOC) with ischemia-reperfusion injury is thought to underlie most, if not all, of these complications.

Based on early observations that deoxygenation or low pH caused red blood cells from patients with HbSS to acquire a sickle shape, it was thought that sickle cell vaso-occlusion resulted solely from obstruction of small blood vessels with these rigid cells. It was later recognized that sickle red cells (sRBC) from SCD patients were aberrantly adhesive.[3]–[5] Multiple receptor-ligand adhesion molecule interactions between sRBC and endothelial cells and sub-endothelial matrix underlie this enhanced adhesiveness.[6]–[12] Furthermore, sRBC populations differ in their adhesiveness (young RBCs or reticulocytes being more adherent than older, more dense cells) and the degree of adhesiveness directly correlates with clinical severity.[4] The model of VOC that emerged involved initial adherence of sRBC reticulocytes to activated endothelium with secondary adherence and capture of more dense, rigid sRBC. Decreased adhesion molecule expression may partially underlie the beneficial effect of hydroxyurea in patients with SCD.[13] More recently, it has been suggested that platelets and leukocytes (specifically neutrophils and monocytes) play a role in acute and chronic morbidity as elucidated by in vitro and in vivo studies.[14]–[18] Heterotypic adhesive events between red cells, platelets, leukocytes, and endothelial cells, are emerging as a model for sickle cell vaso-occlusion.

Several lines of clinical evidence suggest a role for leukocytes in SCD pathogenesis.[19] Elevated leukocyte counts have been associated with increased mortality[20] and acute chest syndrome.[21] The clinical benefit of hydroxyurea may be in part due to a reduction of leukocytes in addition to other mechanisms (increased fetal hemoglobin, decreased red cell adhesion molecule expression, decreased leukocyte adhesion.[22]–[30] In transgenic sickle cell mice, there is increased adherence of leukocytes to endothelium compared with non-sickle control mice following hypoxia or inflammatory stimuli.[14], [31] This adherence, with secondary capture of sickle red blood cells, is the initiating event of VOC in this mouse model.

Turhan and colleagues demonstrated in a sickle mouse that adherent leukocytes bound to post-capillary cremasteric venules.[14] Rolling and capture are mediated by selectins and integrins, respectively. Leukocyte capture onto endothelium stimulates signal transduction mediated conformational changes in leukocyte integrins that further increase competence to bind sickle red blood cells.

The selectins comprise a family of three members mediating adhesion events between blood cells and the endothelium. L-selectin is constitutively expressed on leukocytes and mediates lymphocyte recruitment in lymph nodes and secondary tethers between leukocytes and inactivated venules. Endothelial cells express two selectins, P-selectin which is stored in Weibel-Palade bodies and can be rapidly translocated to the cell surface upon stimulation, and E-selectin whose expression is induced by inflammatory cytokines such as TNF-α or IL-1β. Selectins mediate leukocyte rolling along on the endothelium, allowing circulating leukocytes to rapidly decelerate and come into close contact with chemokines and induce firm adhesion.[32] Transgenic sickle mice deficient in both P-and E- selectins exhibit severe defects in leukocyte adhesion[33], [34] and are protected from VOC.[14] Studies of the individual function of single selectins in a mouse model of SCD have revealed a key role for E-selectin, but not P-selectin, in sending activating signals leading to the upregulation of the β2 integrin, Mac-1, specifically at the leading edge of crawling neutrophils in inflamed venules.[35]

Selectin ligands are composed of a trisaccharide domain common to both sialyl Le^a^ and sialyl Le^x^ (sLe^a/x^)[36]. GMI 1070 is a novel small molecule glycomimetic that was rationally designed to contain both a more potent sLe^a/x^ mimetic and an extended sulfated domain to accommodate the binding requirements of P-and L-selectins and confer drug-like properties to the molecule.[37]

In mouse models containing human sickle hemoglobin, GMI 1070 prevented and reversed VOC when administered well after initiation of the crisis in a clinically relevant treatment protocol.[38]. These data strongly suggest that targeting leukocyte adhesion has therapeutic potential in sickle cell disease.

Based on these animal studies, we performed an open-label, Phase 1 dose-ranging study of IV GMI 1070 in adults with stable SCD. The primary objective was to evaluate the safety of two intravenous (IV) doses of GMI 1070 in adults with sickle cell disease (SCD). The secondary objectives were to evaluate the pharmacokinetics (PK) of GMI 1070; measure the microvascular blood flow before and after infusion with IV GMI 1070; and, determine serial biomarkers of endothelial activation, inflammation, coagulation, and downstream selectin effect in the blood before and after treatment with IV GMI 1070.

## Methods

### Ethics Statement

The Institutional Review Boards of the University of California, Davis; Duke University; and Oakland Children's Hospital approved the study, and all patients gave signed, informed consent. The study was conducted according to the principles of Good Clinical Practice. The protocol for this trial and supporting CONSORT checklist are available as supporting information: see Checklist S1 and Protocol S1.

Eligible subjects were adults aged 18–50 years with an established diagnosis of sickle cell disease/homozygous hemoglobin S (SCD-SS) or sickle cell disease hemoglobin β^0^-thalassemia (SCD-Sβ^0^-thal). Disease activity had to be at the level of the subject's medical baseline, with no evidence of worsening over the last 3 months (e.g. any acute complication of SCD that required unscheduled medical attention or intervention) as determined by the investigator.

This was an open-label, dose-ranging study of IV GMI 1070 in adults with stable SCD. The initial dose level (A) was selected based on preclinical data, and subsequent dose levels could be adjusted in response to in-study data. Dose Level A, consisted of a 20 mg/kg loading dose of IV GMI 1070 (0 hours) followed by a single 10 mg/kg dose of IV GMI 1070 10±1 hours later. The plan was to double the dose if target drug concentrations were not achieved. Because target concentrations were achieved at Level A, no dose escalation was done.

Sampling for PK, biomarkers, and IVM (to assess microvascular blood flow) was performed immediately prior to the loading dose and at specified intervals after the first and second doses of IV GMI 1070.

### Outcome Measures

#### Safety

The primary objective of the study was to evaluate the safety of GMI 1070 in adults with SCD. Safety was assessed by adverse event (AE) reporting, clinical laboratory test results, physical examination, electrocardiogram (ECG), and concomitant medication use. Subjects were to be followed for a total of 28 days after dosing of study drug: a clinic visit at day 7 (± 2 days) and a telephone follow-up at day 28 (± 3 days).

#### Pharmacokinetics

Pharmacokinetic parameters were assessed using plasma concentrations and urinary excretions of GMI 1070. Blood samples for PK were collected before dosing (on the day of dosing) and at the following intervals after the first dose of IV GMI 1070: 30 minutes, 2, 4, 8, 24, and 48 hours. Urine sampling for PK was performed over a 6-hour period starting at the beginning of the first dose. Plasma and urine concentrations of GMI 1070 were measured using validated liquid chromatography/tandem mass spectrometry (LC/MS/MS) assays with lower limits of quantitation of 0.2 µg/mL and 0.5 µg/mL, respectively.

#### Pharmacodynamics

Computer-assisted intravital microscopy (CAIM) was performed at the UC Davis site as per previously published methods[39]–[42] before dosing (on the day of dosing) and at the following intervals following the first dose of IV GMI 1070: 30 minutes, 2, 4, 8, and 24 hours. Plasma sampling for biomarkers of adhesion, inflammation, and downstream selectin effect in the blood was performed before dosing and 4, 8, 24, and 48 hours after administration of the first dose of IV GMI 1070. Analytes measured included: human sE-selectin, sICAM -1, ICAM-3, sPECAM-1, sP-selectin and sVCAM-1 by multiplex bead assay (eBioscience, San Diego, CA; D-dimer by ELISA, tissue factor in plasma and thrombin-antithrombin complexes (TAT), all by ELISA (Seikisui, Stamford, CT); and, surface expression of monocyte β_2_ integrins MAC-1 & LFA-1; and platelet-monocyte aggregates (PMA) by flow cytometry all performed using modifications of previously described techniques[17], [43].

Briefly, samples were pre-treated with or without ADP, labeled with or without anti-CD14-PE, and either anti-cd41a-APC or anti-CD11b-APC, and fixed prior to shipment on ice to the lab. Upon receipt, samples were centrifuged at 1000xg for 3–5 minutes at room temperature to remove the fixative. Samples were then washed once with 0.5ml Hepes Buffered Saline (HBS) and transferred to FACS tubes. One aliquot of the cd14 labeled cells was labeled on site with anti-CD11a-APC for 30 minutes at room temperature. All samples were analyzed on the BD LSR Fortessa Special Order Research Product flow cytometer (Becton Dickinson, San Jose, CA) with DIVA acquisition software and FlowJo analysis software version 8.8.7 (Tree Star Inc. Ashland, OR). Platelet-monocyte aggregates are defined as double positive events (CD14^+^/CD41a^+^) and results are presented as percent of total monocytes (CD14^+^). MAC-1 (CD11b) and LFA-1 (CD11a) results are presented as mean fluorescence intensity (MFI) as compared to the unlabeled cells.

### Analysis Plan

The analysis populations included the following 3 populations:

1. Safety population – all subjects who received at least 1 dose of investigational product (i.e., the loading dose) and had at least 1 post-baseline safety measurement (e.g., vital signs). The safety population was used for summarizing baseline characteristics and safety data.
2. Efficacy population – all subjects who received at least 1 dose of investigational product (i.e., the loading dose) and had at least subsequent 1 CAIM or biomarker measurement. The efficacy population was used for the primary and secondary efficacy analyses (CAIM and biomarkers of adhesion, inflammation, coagulation, and downstream selectin effect).
3. Pharmacokinetic population – all subjects who completed the study and had sufficient data for the PK analyses. The PK population was used to summarize the plasma concentrations of GMI 1070 over time, amount of GMI 1070 excreted in the urine, and PK parameters.

#### Safety

Safety results were summarized descriptively and presented in listings.

#### Pharmacokinetics

GMI 1070 plasma concentration, urinary excretion, and PK parameters were summarized by treatment group using descriptive statistics. Pharmacokinetic parameters were estimated by fitting a 2-compartment IV infusion model to each subject's data. The primary PK parameters estimated in fitting the model included total plasma clearance (CL), intercompartmental clearance (CLD2), volume of the central compartment (V1), and volume of the peripheral compartment (V2). These primary parameters were used to calculate the following secondary PK parameters: maximum observed drug concentration (Cmax), time of maximum drug concentration (Tmax), area under the plasma concentration-time curve to infinity (AUC[inf]), apparent first-order distribution rate constant (α), apparent first-order terminal elimination rate constant (β), elimination half-life (t½), and volume of distribution at steady state (Vss). Urine data were used in conjunction with the PK model to estimate renal clearance for the collection period (CLr). The total amount of drug excreted in the urine during the collection period (Ue) was calculated from the urine concentration of drug (Cu) and the volume of urine collected (Vu), and expressed as milligrams of drug and percentage of administered dose recovered in the urine (Fe). The AUC for the urine collection period was estimated from the PK model, and CLr was calculated as CLr = Ue/AUC. All modeling was done using WinNonlin Professional Version 5.2.

#### Pharmacodynamics

Serial red blood flow velocity from the UC Davis site was calculated, tabulated, and presented in graphical form. Other efficacy (pharmacodynamic) outcome measures included biomarkers of endothelial activation, inflammation, and downstream selectin effect in the blood measured at 4, 8, 24, and 48 hours after administration of the first dose of IV GMI 1070.

Expression levels were compared against pre-treatment, and stratified by HU use. Variables evaluated were the mean changes in microvascular blood flow from baseline to each post-baseline assessment time point, and the changes in biomarkers from baseline to each post-baseline time point.

The hypothesis of no difference between mean baseline value and mean post-baseline value at each time point was assessed using a mixed-effects model. The change from baseline in microvascular blood flow and other biomarkers was modeled longitudinally using a mixed-effects model, with subject as a random effect and time, reader, interaction of time and reader, and baseline value as fixed effects. For continuous variables with repeated measures such as the biomarkers used in this trial, the linear mixed model approach allows use of all the data available.

## Results

**Figure 1:**
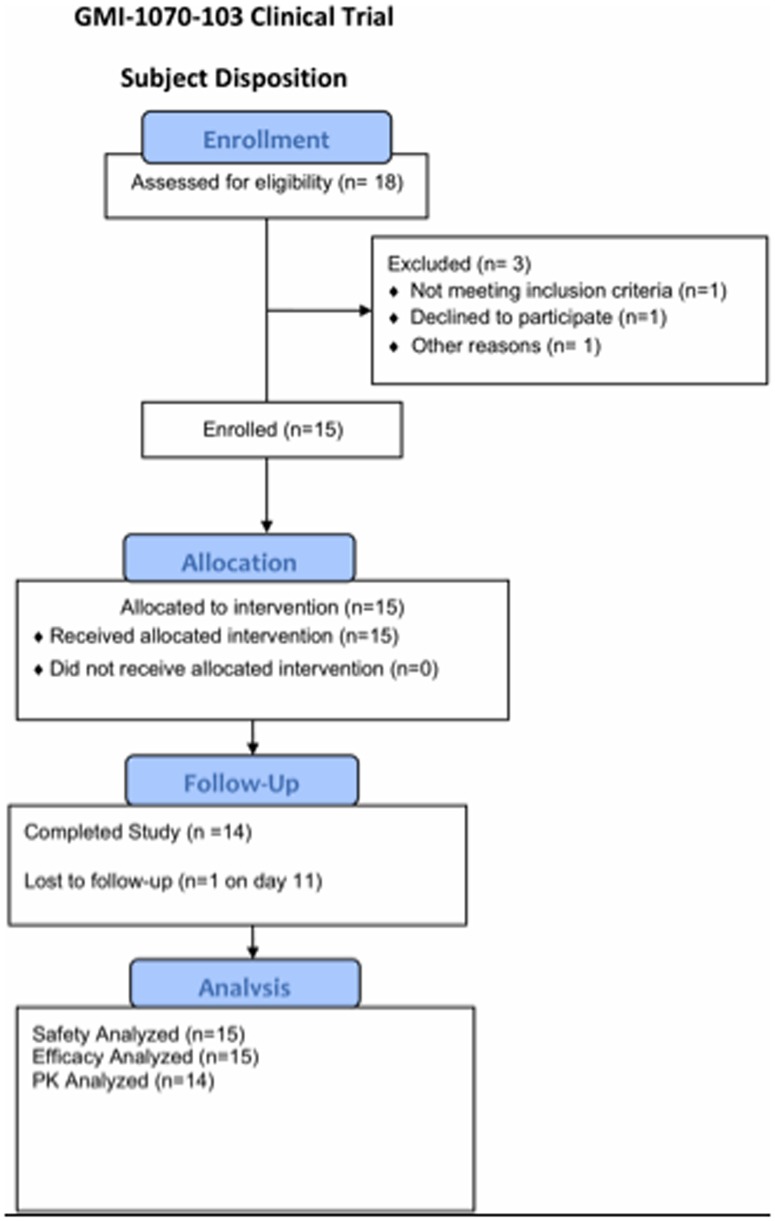
Enrollment Summary.

A total of 15 patients were enrolled (**Table 1** **and** **Figure 1**) between May 28, 2009 (first subject enrolled) to July 6, 2010 (last patient last visit) when adequate data was collected to allow for planning of a Phase 2 study. All were African-American; nine were males, and ages ranged from 19 to 50 years. Thirteen of the 15 had homozygous hemoglobin S disease. Five patients were on a stable dose of hydroxyurea.

**Table 1:** Baseline Demographic Characteristics (Safety Population).

	GMI-1070
	20 mg/kg + 10 mg/kg
Characteristic	N = 15
Statistic or Category	n (%)
**Age (years)**	
n	15
Mean	32.1
SD	10.62
Median	28.0
Min, max	19, 50
**Gender – n (%)**	
Male	9 (60.0)
Female	6 (40.0)
**Baseline weight (kg)**	
n	15
Mean	64.79
SD	9.90
Median	65.5
Min, max	48.3, 90.7
**Genotype – n (%)**	
HbSS	13 (86.7)
HbSβ0-thal	2 (13.3)

Hb  =  hemoglobin; max  =  maximum; min  =  minimum; SD  =  standard deviation; thal  =  thalassemia.

### Safety

A total of 9 (60.0%) subjects reported 16 treatment-emergent adverse events (TEAEs) during the study (**Tables 2** **and** **3**). The most frequently reported were headache (4/15, 26.7%) and vaso-occlusive crisis (VOC) (2/15, 13.3%). Three (20.0%) subjects had TEAEs considered at least possibly related to study drug (headache in 2 subjects and leukocytosis in 1 subject). Except for grade 4 anemia (pre-existing as Grade 4, and worsening slightly while on study) in one subject, all TEAEs were rated grade 1 or 2 in severity. One subject reported a TEAE involving skin. One subject reported grade 1 pruritus of abdomen and back on Day 8, which resolved with no treatment after 3 days.

**Table 2:** Overall Summary of Adverse Events (Safety Population).

	GMI-1070
	20 mg/kg + 10 mg/kg
	N = 15
Type of Event or Subjects with Type of Event	n (%)
Treatment-emergent adverse events	16
Subjects with TEAEs^1^	9 (60.0)
Treatment-emergent SAEs	0
Subjects with treatment-emergent SAEs^1^	0 (0)
Treatment-related TEAEs	3
Subjects with treatment-related TEAEs^1^	3 (20.0)
Treatment-related SAEs	0
Subjects with treatment-related SAEs^1^	0 (0)
Subjects with TEAEs by severity^2^	
Grade 1	5 (33.3)
Grade 2	3 (20.0)
Grade 3	0
Grade 4	1 (6.7)^3^
Subjects with TEAEs leading to discontinuation	0
Deaths	0

SAE  =  serious adverse event; TEAE  =  treatment-emergent adverse event.

1 Subjects who experienced 1 or more adverse event were counted once.

2 If a subject experienced more than 1 adverse event, the subject was counted only once for the worst (or maximum) severity.

3 Event was grade 4 anemia (hemoglobin 5.9 g/dL), which was not considered serious by the investigator.

**Table 3:** Treatment-emergent Adverse Events by System Organ Class and Preferred Term (Safety Population).

	GMI-1070
	20 mg/kg + 10 mg/kg
System Organ Class	N = 15
Preferred Term	n (%)
**Any treatment-emergent adverse event**	9 (60.0)
**Nervous system disorders**	4 (26.7)
Headache	4 (26.7)
**Blood and lymphatic system disorders**	2 (13.3)
Anemia	1 (6.7)
Leukocytosis	1 (6.7)
**Congenital, familial, and genetic disorders**	2 (13.3)
Sickle cell anemia with crisis^1^	2 (13.3)
**Gastrointestinal disorders**	1 (6.7)
Vomiting	1 (6.7)
**General disorders and administration site conditions**	1 (6.7)
Infusion site pain	1 (6.7)
**Metabolism and nutrition disorders**	1 (6.7)
Hypokalemia	1 (6.7)
**Musculoskeletal and connective tissue disorders**	1 (6.7)
Arthralgia	1 (6.7)
**Respiratory, thoracic, and mediastinal disorders**	1 (6.7)
Cough	1 (6.7)
Oropharyngeal pain	1 (6.7)
**Skin and subcutaneous tissue disorders**	1 (6.7)
Pruritus	1 (6.7)

Events are listed in descending order of frequency of preferred terms, with grouping by system organ class.

If a subject experienced more than 1 preferred term of adverse event, the subject was counted only once for that preferred term.

Urinary tract infection reported for one subject was not included because the event occurred on study Day -3.

1 New vaso-occlusive crisis.

No deaths, serious adverse events, or TEAEs leading to discontinuation were reported during the study.

Eight (53.3%) subjects reported 10 TEAEs involving pain. These included the headache and VOC (13 and 23 days after infusion) already mentioned, and 1 (6.7%) subject each with infusion site pain, arthralgia, and oropharyngeal pain. All of the events were grade 1 in severity except for grade 2 headache in one subject and grade 2 VOC in another. The headache began on study Day 1 and was considered probably related to study drug. A headache in another subject (grade 1, onset Day 2) was considered possibly related. The remaining pain events were considered unlikely related (7 subjects) or unrelated (oropharyngeal pain), with onset days ranging from Day 1 to Day 24.

No notable trends in vital signs were observed during the study. For each body system, the majority (≥73.3%) of subjects had no changes in physical examination findings, and those with changes had findings that were not considered clinically significant.

Mean values for aspartate aminotransferase (AST) and lactate dehydrogenase (LDH) were elevated at baseline and showed decreases at subsequent time points, but they remained close to or above the upper limit of reference range throughout the study. At the Day 7 Visit, the mean (SD) changes in AST and LDH from baseline were -13.9 (24.4) U/L and -98.9 (134.7) U/L, respectively, and the mean (SD) observed values were 41.9 (14.6) U/L and 629.9 (378.2) U/L. Mean values for total bilirubin were also elevated at baseline (3.76 [3.08] mg/dL), and subsequent values were generally similar to those at the Baseline Visit. The elevations in AST, LDH, and total bilirubin were consistent with expected abnormalities in individuals with SCD.

Hematocrit and hemoglobin values for the safety population showed small mean increases from baseline at the Day 2 and 3 Visits. At the Day 3 Visit, the mean (SD) changes in hematocrit and hemoglobin from baseline were 1.56 (1.80)% and 0.35 (0.62) g/dL, respectively.

**Figure 2:**
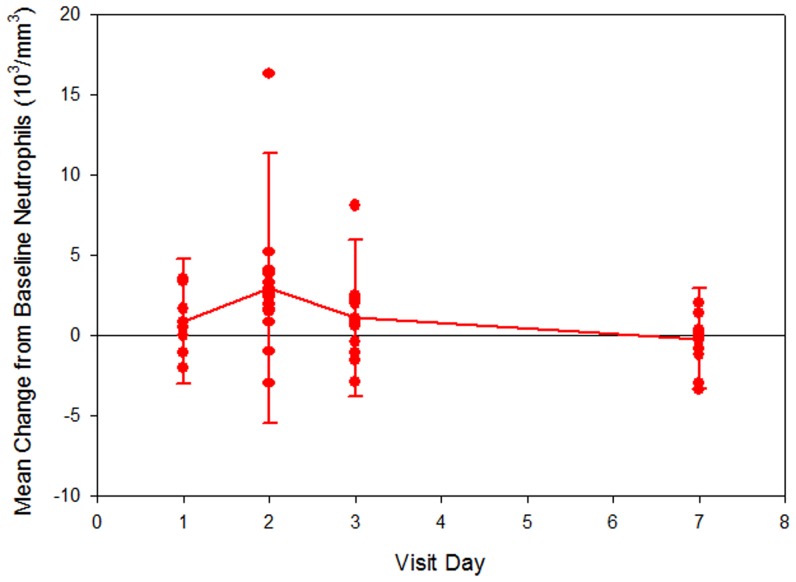
Change in absolute neutrophil count from baseline.

Moderate increases in neutrophil and total white blood cell (WBC) counts were observed at the Day 2 Visit (24 hours), when the mean (SD) values were 7.5 (5.5)×10^3^/mm^3^ and 11.6 (6.6)×10^3^/mm^3^, respectively (Figures **2** **–** **3**). The corresponding changes from baseline were significant: 3.2 (4.5)×10^3^/mm^3^ (p-value <0.001) for neutrophil count and 2.3 (4.8)×10^3^/mm^3^ (p-value = 0.013) for WBC count. Mean values for neutrophil and WBC counts returned to baseline values by Day 7. This pattern of increase in neutrophil and WBC counts at the Day 2 Visit was observed in the subjects who were not taking hydroxyurea during the study. There was no significant increase in neutrophil counts at 24 hours in the patients taking hydroxyurea group (p = 0.2). However, these data should be interpreted with caution given the small sample size.

**Figure 3:**
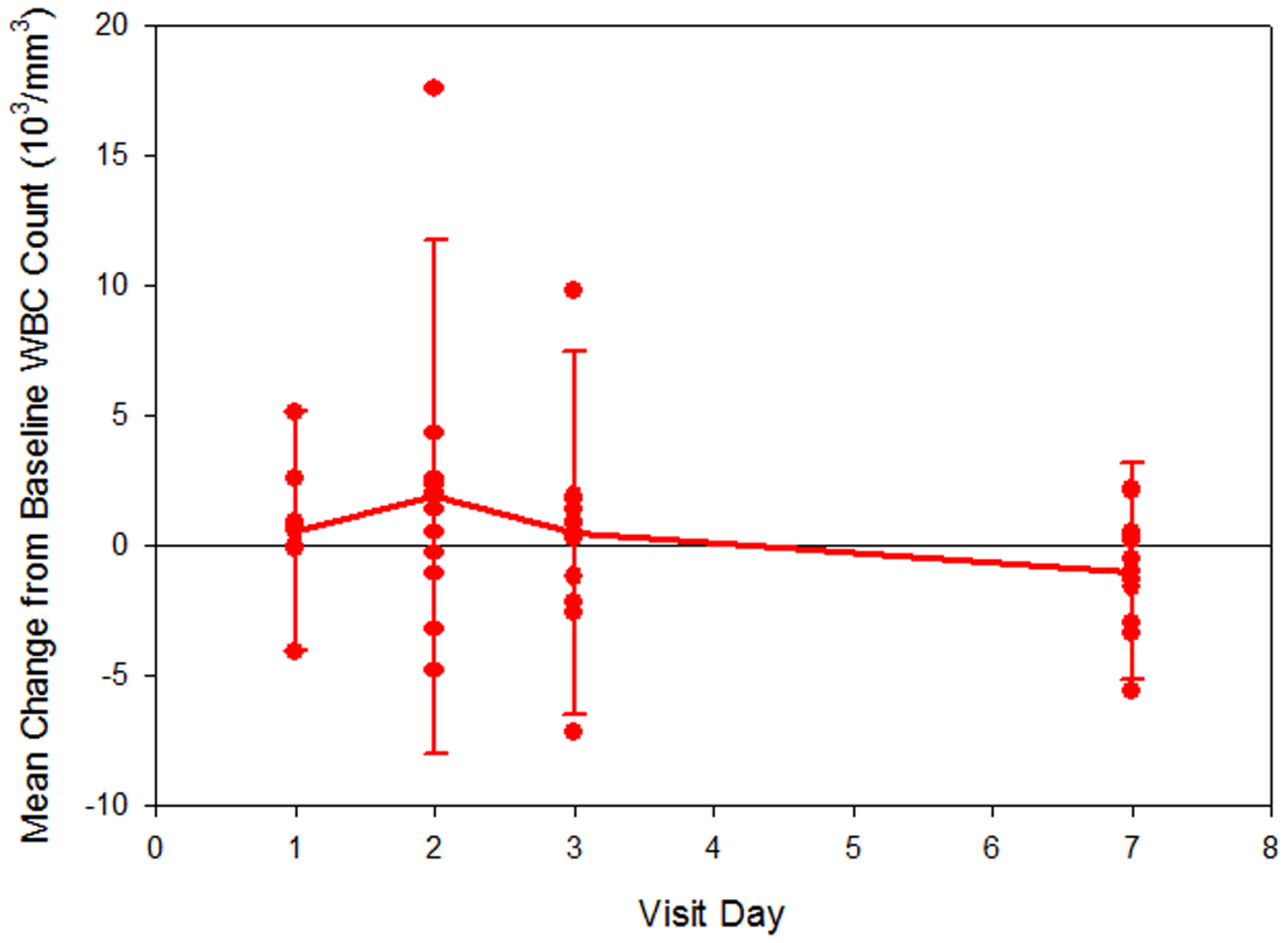
Change in total white blood cell count (WBC) from baseline.

**Figure 4:**
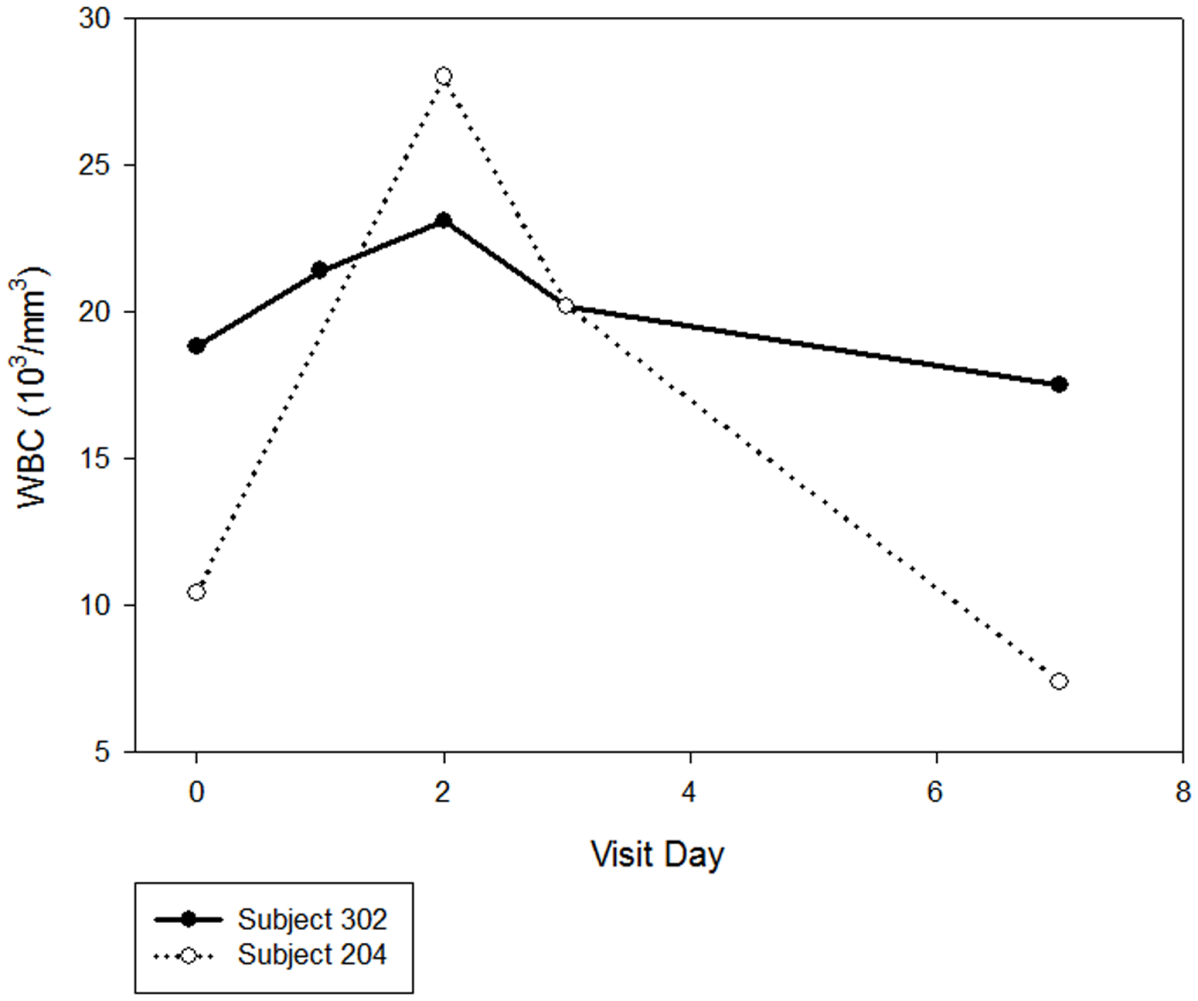
Change in total white blood cell count (WBC) in the two subjects with marked leukocytosis.

The high sensitivity C-reactive protein (hsCRP) level increased from 4.3 (4.1) mg/L at the Baseline Visit to 9.0 (15.7) mg/L at the Day 3 Visit, and then decreased to a level near baseline (4.2 [5.4] mg/L) at the Day 7 Visit. Two subjects had notable increases in hsCRP during the study, with peak values of 50.4 and 40.7 mg/L, respectively, at the Day 3 Visit. In both subjects, the increases were associated with significant increases in WBC count, and in one, with a pre-existing asymptomatic urinary tract infection (**Figure 4**). Neither subject had symptoms of infection or increased pain after treatment with GMI 1070.

Clinically significant laboratory test results (reported as adverse events and requiring clinical follow-up) were reported in 3 subjects. One subject had a hemoglobin value of 5.9 g/dL (Day 7 Visit), decreased from a baseline of 6.0 g/dL. Another subject had a WBC count of 28.0×10^3^/mm^3^ and 20.2×10^3^/mm^3^ (Day 2 and 3 Visits, respectively), and remained asymptomatic during this time. Another subject had an elevated LDH (476 U/L at baseline); decreased serum potassium (2.6 mEq/L at Day 3 Visit); increased WBC count (18.8, 21.4, 23.1, 20.2, and 17.5×10^3^/mm^3^ at the Baseline and Day 1 [post dose], 2, 3, and 7 Visits, respectively); increased neutrophil count (12.8, 14.5, and 11.6×10^3^/mm^3^ at Day 1 [post dose], 2, and 3, respectively); and several microscopic urinalysis variables consistent with a pre-existing asymptomatic urinary tract infection.

### Pharmacokinetic Results

**Figure 5:**
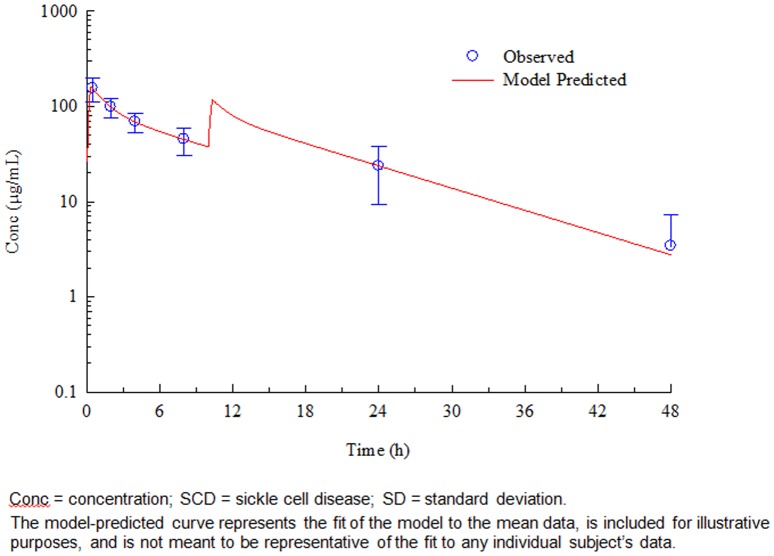
Observed and model-predicted plasma concentrations of GMI-1070 after intravenous infusion.

Fourteen (14) of the 15 subjects had sufficient data for PK analysis. The PK of GMI 1070 in subjects with SCD was consistent with a 2-compartment IV infusion model, and the estimated clearances, volumes of distribution, T_1/2_ (7.7 hours) and CLr (19.6 mL/hour/kg) were consistent with those observed previously in healthy volunteers.[44]–[46] The model-predicted plasma GMI 1070 concentrations for all 14 subjects in the PK population showed excellent agreement with the observed concentrations; this is illustrated with the mean data in Figure **5**. These results also demonstrated that the use of a loading dose achieves immediate steady-state plasma concentrations of GMI 1070 in individuals with SCD and that the use of this dose level (20 mg/kg loading dose) followed by a 10 mg/kg maintenance dose achieves plasma concentrations expected to have activity in this population.

### Pharmacodynamic Results

**Figure 6:**
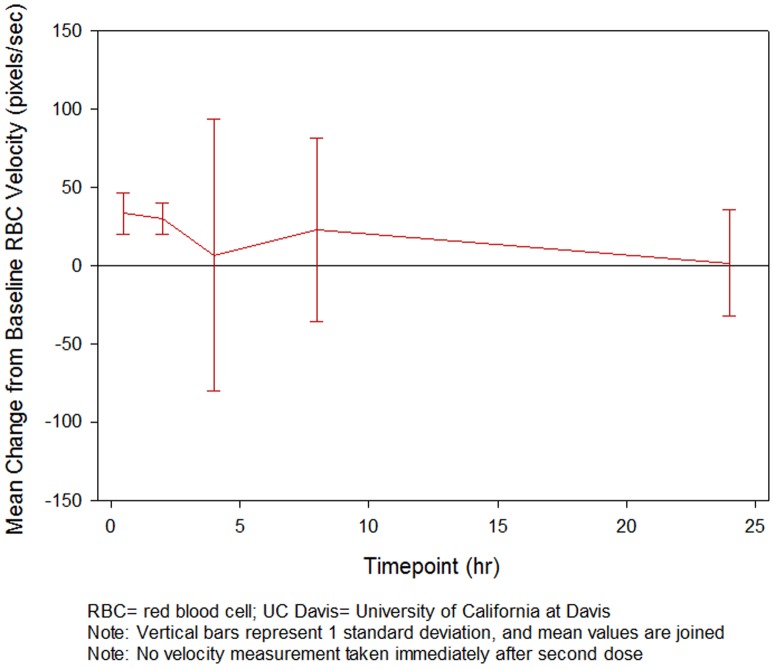
Mean change in RBC velocity as measured by computer-assisted intravital microscopy.

At the UC Davis site, the mean observed RBC velocity showed an increase over the baseline value at 30 minutes, 2 hours, 4 hours, 8 hours and 24 hours after dosing. The largest mean (SD) increase in RBC velocity from baseline occurred 30 minutes post dosing, with a value of 33.30 (13.52) pixels/sec (Figure **6**). All four subjects had detectable plasma levels of GMI 1070, consistent with plasma levels predicted by the PK model, during velocity measurements. This trend toward an increase in RBC velocity from baseline may indicate improved blood flow in small blood vessels, but it did not reach statistical significance (all p-values >0.05).

Statistically significant reduction of multiple biomarkers of adhesion, activation, and coagulation was observed after treatment with GMI 1070. Soluble adhesion markers were reduced after 8 hrs (soluble E-selectin), and after 4 and 8 hrs (soluble P-selectin; ICAM-1). Tissue factor (TF) was reduced at 4 and 8 hrs (**Table 4**). Thrombin-antithrombin complex (TAT) levels were reduced at all time points (4 hr, 8 hr, 24 hr, 48 hr). The percentage of PMA was reduced at 8 hrs. The expression of MAC-1 and LFA-1 was reduced at all time points. When HU use was considered (HU–No; HU–Yes), the levels of sEsel, sPsel, ICAM-1, TF, TAT, MAC-1, and neutrophil counts were lower and more variable at baseline in the HU-No group, significantly so for ICAM-1 (p = 0.048) and MAC-1 (p = 0.001). After GMI 1070, significant reduction from baseline was seen in both groups: in the HU-No group for ICAM-1, TF, PMA, LFA-1, and in the HU-Yes group for sEsel, MAC-1, TF, TAT. However, again due to the small sample size caution should be exercised in interpreting these results.

**Table 4:** Biomarker Results

		4 Hours			8 Hours			24 Hours			48 Hours		
Biomarker	Baseline Level (SD)	LS Mean (CI)	Change from Baseline, LS Mean (CI)	p-value	LS Mean (CI)	Change from Baseline, LS Mean (CI)	p-value	LS Mean (CI)	Change from Baseline, LS Mean (CI)	p-value	LS Mean (CI)	Change from Baseline, LS Mean (CI)	p-value
**sE-sel (ng/mL)**	117.2 (48.6)	104.0 (93.1, 115.0)	−10.53 (−21.4, 0.4)	0.058	99.2 (89.7, 108.7)	−15.4 (−24.9, −5.9)	**0.004**	105.5 (95.5, 115.4)	−9.11 (−19.1, 0.8)	0.070	109.4 (99.6, 119.1)	−5.21 (−14.9, 4.5)	0.272
**sP-sel (ng/mL)**	180.4 (154.0)	122.6 (94.1, 151.1)	−31.2 (−59.6, −2.7)	**0.034**	122.9 (96.1, 149.7)	−30.9 (−57.7, −4.1)	**0.028**	134.0 (106.7, 161.3)	−19.8 (−47.1, 7.5)	0.141	138.5 (111.4, 165.5)	−15.3 (−42.3, 11.7)	0.241
**ICAM-1 (ng/mL)**	239.6 (177.8)	195.2 (179.1, 211.3)	−19.4 (−35.5, −3.3)	**0.021**	190.6 (175.7, 205.4)	−24.1 (−38.9, −9.3)	**0.004**	203.3 (188.1, 218.5)	−11.3 (−26.5, 3.9)	0.133	202.5 (187.5, 217.6)	−12.1 (−27.1, 2.9)	0.105
**TF (pg/mL)**	466.7 (313.4)	323.8 (239.1, 408.4)	−120.5 (−205.1, −35.8)	**0.009**	364.1 (283.9, 444.2)	−80.2 (−160.3, −0.1)	**0.050**	374.9 (293.5, 456.4)	−69.3 (−150.8, 12.1)	0.087	374.6 (293.8, 455.4)	−69.6 (−150.4, 11.2)	0.083
**TAT (ng/mL)**	145.4 (94.5)	40.3 (0.7, 79.8)	−104.0 (−143.5, −64.5)	**<0.001**	83.6 (54.9, 112.2)	−60.7 (−89.3, −32.0)	**<0.001**	63.1 (31.6, 94.6)	−81.2 (−112.7, −49.7)	**<0.001**	93.2 (63.2, 123.3)	−51. 0 (−81.1, −21.0)	**0.002**
**MAC-1 (MFI)**	3278.5 (1302.4)	2271.5 (1559.3, 2983.8)	−1235.7 (−1948.0, −523.4)	**0.002**	2475.7 (1999.3, 2952.1)	−1031.5 (−1507.9, −555.1)	**0.001**	2742.8 (2227.5, 3258.0)	−764.5 (−1279.7, −249.1)	**0.008**	2641.6 (2146.6, 3136.7)	−865.6 (−1360.6, −370.6)	**0.004**
**LFA-1 (MFI)**	815.3 (271.8)	571.6 (386.3, 757.0)	−294.3 (−479.7, −109.0)	**0.004**	695.8 (593.2, 798.5)	−170.1 (−272.8, −67.4)	**0.004**	705.0 (584.8, 825.1)	−161.0 (−281.1, −40.8)	**0.012**	704.0 (593.6, 814.5)	−161.9 (−272.3, −51.5)	**0.008**
**PMA (%)**	82.1 (20.2)	72.8 (48.9, 96.7)	−9.2 (−33.1, 14.7)	0.425	61.0 (42.3, 79.7)	−20.9 (−39.6, −2.2)	**0.033**	75.3 (55.8, 94.9)	−6.6 (−26.2, 13.0)	0.464	76.9 (57.8, 96.0)	−5.0 (−24.1, 14.1)	0.559

## Discussion

The primary objective of this study was to evaluate the safety of two intravenous doses of GMI 1070 in clinically stable adults with SCD. No serious adverse events were reported, and no subject discontinued the study because of an adverse event. All adverse events except one were grade 2 or less in severity. The single grade 4 event was anemia, which was considered to be non-serious by the investigator and consistent with underlying sickle cell anemia. Three subjects reported TEAEs considered possibly or probably related to study drug, which included headache (2 subjects) and leukocytosis (1 subject). Laboratory values showed no notable trends of worsening over time. Overall no serious concerns were identified in this study with regard to the safety of GMI 1070.

Moderate and significant mean increases in neutrophil (p-value <0.001) and WBC (p-value = 0.013) counts were observed at the Day 2 Visit (24 hours), followed by a return to values similar to baseline by Day 7. Thus, administration of GMI 1070 was associated with neutrophilia and leukocytosis that were moderate in intensity, temporally associated with plasma levels of the drug, and reversible after elimination of the drug from the plasma. These findings are consistent with the expected anti-adhesive effect of selectin inhibition, and likely represent release of adherent leukocytes from the vascular endothelium into the peripheral circulation. Similar increases in neutrophil and WBC count were not observed in the subgroup of subjects who took hydroxyurea during the study, although small fluctuations in these counts were seen in individuals.

The WBC and neutrophil results from this study suggests that leukocyte adhesion can be inhibited at pharmacological levels in patients with sickle cell disease, although whether this will be enough to alleviate established vascular occlusion in patients presenting in VOC remains to be seen. Leukocytosis could theoretically have detrimental effect in patients with sickle cell disease, such as increased incidence of acute chest syndrome or infection. These potential adverse effects will have to be carefully followed in further clinical studies.

A secondary study objective was to evaluate the PK of two IV doses of GMI 1070 in adults with SCD. The plasma concentrations were concordant with a 2-compartment IV infusion model, and the estimates for clearances, volumes of distribution, t½, and CLr were consistent with those observed previously in healthy volunteers. The model-predicted plasma GMI 1070 concentrations for all 14 subjects in the PK population showed excellent agreement with the observed concentrations. The results support the use of a 20 mg/kg loading dose followed by a 10 mg/kg dose to rapidly reach and maintain plasma concentrations of GMI 1070 expected to be effective in individuals with SCD.

Another secondary objective was to evaluate microvascular blood flow before and after administration of IV GMI 1070. Previous work by our group has demonstrated that acute changes in blood flow in response to various therapies are detectable by this method.[39], [47] Although subjects showed mean increases in RBC velocity compared to baseline at 30 minutes and 2, 4, 8 and 24 hours post dosing, which may indicate improved blood flow, the trend toward an increase in RBC velocity did not reach statistical significance. However, the sample size (n = 4) may have precluded finding significant differences. The timing was consistent with observed WBC/ANC changes, suggesting pharmacologic response of increased WBC and ANC may correspond with improved microvascular flow.

The effects of GMI 1070 were evaluated on biomarkers known to be elevated in sickle cell disease and mechanistically affected by the target molecule, E-selectin. Markers of leukocyte activation (MAC-1; LFA-1); platelet activation (soluble P-selectin; platelet-monocyte aggregates or PMA); vascular inflammation (soluble E-selectin; soluble ICAM-1); monocyte activation (PMA); and coagulation system activation (tissue factor and thrombin-antithrombin complexes) were serially measured. At some time point between 4 and 48 hours after intravenous infusion of GMI 1070, there were significant decreases in all these various markers consistent with downstream inhibition of cellular activation and an overall anti-inflammatory effect. In some individuals these changes persisted at a time when drug plasma levels were below 10 µg/mL. As the pathophysiology of VOC involves both leukocyte adhesion and downstream inflammation,[15], [16], [48] these findings provide further rationale for use of GMI 1070 in VOC.

When administered to adults with SCD at steady state, the pan-selectin inhibitor GMI 1070 had safety and PK profiles similar to those seen in healthy volunteers. This study provides mechanism-based evidence of effect of GMI 1070 in this population and supports further evaluation of the drug in the treatment of vaso-occlusive crisis. A prospective randomized double-blinded Phase 2 trial of GMI 1070 in patients with SCD and VOC has been conducted based on results of this Phase 1 study.

## Supporting Information

Checklist S1 **CONSORT Checklist.**
(DOC)

Protocol S1 **Trial Protocol.**
(PDF)
