# Correction: Phase 1 Study of the E-Selectin Inhibitor GMI 1070 in Patients with Sickle Cell Anemia

**DOI:** 10.1371/journal.pone.0111690

**Published:** 2014-10-17

**Authors:** The PLOS ONE Staff

The affiliation for the fourth author is incorrect. Marilyn J. Telen is not affiliated with #6 but with Duke University Medical Centre, Durham, North Carolina, United States of America.
