# Supplementary material for: Phase 1 Study of the E-Selectin Inhibitor GMI 1070 in Patients with Sickle Cell Anemia
Source: PLoS One. 2014 Jul 2;9(7):e101301. doi: 10.1371/journal.pone.0101301 (PMC4079300; doi:10.1371/journal.pone.0101301)
Supplement: Protocol S1 — Trial Protocol. (PDF) [file pone.0101301.s002.pdf]

PHASE 1/2 STUDY OF THE SAFETY, PHARMACOKINETICS, AND MICROVASCULAR EFFECT OF TITRATING DOSES OF INTRAVENOUS GMI-1070, A PAN-SELECTIN INHIBITOR, IN ADULTS WITH SICKLE CELL DISEASE

Information in this protocol is CONFIDENTIAL and should not be disclosed, other than to those directly involved in the execution or the ethical review of the study, without written authorization from GlycoMimetics, Inc. (GMI).

**PROTOCOL NUMBER:**	GMI-1070-103
**PROTOCOL VERSION AND DATE:**	Version 1.3 dated 27 October 2009
**PREVIOUS PROTOCOL VERSIONS:**	Version 1.2 incorporating Amendment 2, dated 24 August 2009<br><br>Version 1.1, Amendment 1 dated 04 May 2009<br><br>Version 1.0, Original dated 17 February 2009
**DRUG:**	GMI-1070 administered intravenously in multiple doses
**SPONSOR:**	GlycoMimetics, Inc.<br>101 Orchard Ridge Drive, Suite 1E<br>Gaithersburg, MD 20878<br>Telephone (240) 243-1230<br>Fax (240) 599-7680
**INVESTIGATORS:**	Lori Styles, MD<br>Hematologist/Oncologist<br>Children's Hospital Oakland Research Institute<br>5700 Martin Luther King Jr. Way<br>Oakland, CA 94609<br>Telephone (510) 428-3553<br>email: lstyles@mail.cho.org

INVESTIGATORS (continued):

Marilyn J. Telen, MD
Division Chief, Division of Hematology
Duke Comprehensive Sickle Cell Center
Duke University Medical Center
Box 2615
Durham, NC 27710
919-684-5378
email: telen002@mc.duke.edu

Ted Wun, MD, FACP
Professor of Medicine, Pathology and Laboratory Medicine
University of California at Davis
Clinical and Translational Sciences Center
Division of Hematology and Oncology, #6165
4501 X Street, Suite 3016
Sacramento, CA 95817
Telephone: (916) 734-3771
email: twun@ucdavis.edu

CLINICAL RESEARCH ORGANIZATION:

Rho, Inc.
6330 Quadrangle Dr., Ste. 500
Chapel Hill, NC 27517

DATA MANAGEMENT:

Nancy Darrow
Amarex Clinical Research
20201 Century Blvd, 4th floor
Germantown, MD 20874
240-454-2874

EMERGENCY CONTACT LIST

MEDICAL MONITOR:

Helen Thackray, MD
Vice President, Clinical Development
GlycoMimetics, Inc.
101 Orchard Ridge Drive, Suite 1E
Gaithersburg, MD 20878
Telephone (240) 243-1230
Fax (240) 599-7680
Email: hthackray@glycomimetics.com

CLINICAL RESEARCH ASSOCIATE (CRA):

Carmen Wilberg
7313 Castle Drive
Dublin, CA 94568
Telephone (925) 828-7564
Fax: (925) 828-9017
Email: cmwilberg@comcast.net

CRO SAE HOTLINE:

SAE Hotline (888) 746-7231
SAE Fax (888) 746-3293
Victoria C. Williams, PharmD
Sr. Product Safety Scientist
Rho, Inc.
Telephone (919) 595-6237
Email: Victoria_Williams@rhoworld.com

PROTOCOL SIGNATURE PAGE

PROTOCOL NUMBER: GMI-1070-103

PROTOCOL TITLE: Phase 1/2 Study of the Safety, Pharmacokinetics, and Microvascular Effect Of Titrating Doses of Intravenous GMI-1070, a Pan-selectin Inhibitor, in Adults with Sickle Cell Disease

VERSION/DATE: Version 1.3 27 October 2009

SPONSOR'S APPROVAL:

This protocol has been approved by GlycoMimetics, Inc. (GMI).

SPONSOR'S RESPONSIBLE MEDICAL OFFICER:

October 27, 2009

Helen Thackray, MD
Vice President, Clinical Development
GlycoMimetics, Inc.

Date

INVESTIGATOR'S AGREEMENT:

I have read and reviewed this protocol (GMI-1070-103), and I agree to conduct this trial according to this protocol, to comply with its requirements subject to ethical and safety considerations, and to conduct the trial in accordance with International Conference on Harmonisation guidelines on Good Clinical Practice and with applicable local and federal regulatory requirements.

(Signature) **Date**

(Print name)

TABLE OF CONTENTS

EMERGENCY CONTACT LIST 3

PROTOCOL SIGNATURE PAGE 4

TABLE OF CONTENTS 5

LIST OF TABLES 7

LIST OF FIGURES 7

LIST OF APPENDICES 8

ABBREVIATIONS AND DEFINITIONS 9

STUDY SYNOPSIS 11

STUDY SCHEDULE 16

1 BACKGROUND INFORMATION 17

1.1 SICKLE CELL DISEASE 17
1.2 VASCULAR OCCLUSION IN THE PATHOGENESIS OF SICKLE CELL COMPLICATIONS 17
1.3 INTRAVITAL MICROSCOPY 20
1.4 TREATMENT OF VASO-OCCLUSIVE CRISIS 20
1.5 NONCLINICAL INFORMATION 21
1.5.1 Pharmacology *21*
1.5.2 Pharmacokinetics *22*
1.5.3 Toxicology *22*
1.6 CLINICAL INFORMATION 22
1.6.1 Safety *23*
1.6.2 Pharmacokinetics *23*

2 STUDY OBJECTIVES AND PURPOSE 24

2.1 RATIONALE FOR THIS STUDY 24
2.2 STUDY OBJECTIVES 25
2.2.1 Primary *25*
2.2.2 Secondary *25*

3 STUDY DESIGN 25

4 SELECTION AND WITHDRAWAL OF SUBJECTS 28

4.1 NUMBER OF SUBJECTS 28
4.2 INCLUSION CRITERIA 28
4.3 EXCLUSION CRITERIA 28
4.4 WITHDRAWAL OF SUBJECTS 29

5 STUDY AND CONCOMITANT TREATMENTS 30

5.1 GMI-1070 30
5.1.1 Allocation of Subjects to Treatment 31
5.1.2 Labeling, Packaging, Storage, and Handling 31
5.1.3 Compliance and Drug Accountability 31
5.2 CONCOMITANT MEDICATIONS 32

6 STUDY PROCEDURES 32

6.1 STUDY SCHEDULE 32
6.1.1 Screening Visit 32
6.1.2 Day of GMI-1070 Administration 33
6.2 STUDY EVALUATIONS AND PROCEDURES 33
6.2.1 Safety 33
6.2.2 Non-Safety Sampling 35
6.2.3 Clinical Effect 36
6.2.4 Pharmacokinetic Samples 36
6.2.5 Review to Determine Next Dose Level 36

7 ADVERSE AND SERIOUS ADVERSE EVENTS 37

7.1 SAFETY MONITORING 37
7.2 REPORTING 38
7.3 SERIOUS ADVERSE EVENT 39
7.4 DOSE LIMITING TOXICITIES AND STUDY STOPPING RULES 41

8 DATA COLLECTION AND DATA MONITORING 42

8.1 DATA QUALITY ASSURANCE 42
8.2 CASE REPORT FORM AND SOURCE DOCUMENTATION 42
8.3 DATA MANAGEMENT 42
8.4 DATA MONITORING 42

9 STATISTICAL ANALYSIS 43

9.1 ANALYSIS POPULATIONS 43
9.2 STATISTICAL METHODS 43

9.2.1 Subject Disposition 43
9.2.2 Demographics and Baseline Characteristics 44
9.2.3 Efficacy 44
9.2.4 Safety Data 44
9.2.5 Pharmacokinetic Analysis 45
9.2.6 Vital Signs and Laboratory Parameters 46
9.2.7 Physical Examinations 47
9.2.8 Investigational Product Interim Analysis 47
9.3 STATISTICAL CONSIDERATIONS 47
9.3.1 Missing Data 47
9.3.2 Sample Size 47

10 STUDY ADMINISTRATION 47

10.1 DIRECT ACCESS TO SOURCE DATA/DOCUMENTS 47
10.2 QUALITY ASSURANCE/QUALITY CONTROL 48
10.3 DATA HANDLING AND RECORD KEEPING 48
10.4 ETHICAL CONSIDERATIONS 48
10.4.1 Basic Principles 48
10.4.2 Institutional Review Board 49
10.4.3 Protocol Amendments 49
10.5 ADDITIONAL STUDY SITES 49

11 REFERENCES 50

12 APPENDICES 52

LIST OF TABLES

Table 1: Schedule of Assessments 16
Table 2: Clinical Laboratory Evaluations 34
Table 3: Data Reviewed to Determine Next Dose Level 36

LIST OF FIGURES

Figure 1: Sickled Red Blood Cells and Neutrophils Occlude Vasculature[4] 19
Figure 2: IV GMI-1070 Pharmacokinetics with Single Dose in Volunteers: 2-40 mg/kg 24
Figure 3: Study Titration Flow Chart 27

LIST OF APPENDICES

APPENDIX 1 WHO COMMON TOXICITY CRITERIA 53

APPENDIX 2 DOCUMENT HISTORY - SUMMARY OF PROTOCOL AMENDMENTS 59

ABBREVIATIONS AND DEFINITIONS

Abbreviation	Definition
AE	Adverse event
ALT	Alanine transaminase (SGPT)
AST	Aspartate transaminase (SGOT)
AUC	Area under the curve
bid	Twice daily
BMI	Body mass index
CHF	Congestive heart failure
CFR	Code of Federal Regulations
CL	Clearance
CL_r	Renal clearance
C_{max}	Maximum concentration
CRC	Clinical Research Center
C_{urine}	Concentration excreted in urine during each collection interval
CNS	Central nervous system
CRF	Case report form
CRO	Contract research organization
ECG	Electrocardiogram
GCP	Good Clinical Practice
GMI-1070	GMI-1070 Injection, 30 mg/mL
Hb	Hemoglobin
hsCRP	High sensitivity C-reactive protein
ICAM	Intracellular adhesion molecules
ICH	International Conference on Harmonisation
IRB	Institutional Review Board
IV	Intravenous
IVM	Intravital microscopy
kg	Kilogram
LDH	Lactate dehydrogenase
NOAEL	No observed adverse effect level
NSAID	Nonsteroidal anti-inflammatory drug
PFT	Pulmonary function test

Abbreviation	Definition
PK	Pharmacokinetic
RBC	Red blood cells
SAE	Serious adverse event
SAP	Statistical Analysis Plan
SCD	Sickle cell disease
SCD-Sβ^+-thal	Sickle cell disease Hb β^+-thalassemia
SCD-Sβ^0-thal	Sickle cell disease Hb β^0-thalassemia
SCD-SC	Sickle cell disease Hb C
SCD-SS	Sickle cell disease Hb S
SGOT	Aspartate transaminase
SGPT	Alanine transaminase
$t_{½}$	Apparent terminal half-life
TEAE	Treatment-emergent adverse event
t_{max}	Time to maximum concentration
US	United States
VCAM	Vascular cell adhesion molecules
VOC	Vaso-occlusive crisis
V_z	Apparent volume of distribution at the terminal phase
WBC	White blood cell
WHO	World Health Organization
WNL	Within normal limits

STUDY SYNOPSIS

**Protocol number:** GMI-1070-103	**Study drug:** Intravenous GMI-1070
**Title of the study:**<br>Phase 1/2 study of the safety, pharmacokinetics, and microvascular effect of titrating doses of intravenous GMI-1070, a pan-selectin inhibitor, in adults with sickle cell disease	
**Clinical phase:** 1/2	
**Sites:** 3 in the United States (US)	
**Objectives:**<br>**Primary Objective:**<br>• Evaluate the safety of multiple intravenous (IV) doses of GMI-1070 in adults with sickle cell disease (SCD).<br>**Secondary Objectives:**<br>• Evaluate the pharmacokinetics (PK) of multiple IV doses of GMI-1070 in adults with SCD.<br>• Evaluate the microvascular blood flow before and after treatment with IV GMI-1070 in adults with SCD.<br>• Evaluate biomarkers of adhesion, inflammation, and downstream selectin effect in the blood before and after treatment with IV GMI-1070 in adults with SCD.	
**Methodology:**<br>This will be an open-label, dose titration study of IV GMI-1070 in adults with stable SCD. Subjects will receive IV GMI-1070 at dose levels based on findings in earlier human studies. Study drug will be administered in the Clinical Research Center (CRC), day hospital, or equivalent clinical setting.<br><br>Titration for this study may be done either up or down and not necessarily in a step-wise fashion. At the beginning of the study, 4 subjects at each site will receive Dose Level A, which consists of a 20 mg/kg loading dose of IV GMI-1070 followed by a single 10 mg/kg dose of IV GMI-1070. Thus, subjects will receive a total of 2 doses: 1 loading dose (20 mg/kg) in the morning at 0 hours, and 1 single dose (10 mg/kg) in the evening at 10 ± 1 hours.<br><br>After these first 4 subjects at each site have been treated, safety, PK, and blood flow data from this first group will be reviewed by the investigator and the Medical Monitor. After this review, a site-specific decision will be made to do 1 of the following:<br>• Enroll 4 more subjects at this dose level (continue at Dose Level A), OR<br>• Enroll 4 subjects at the higher dose level (Dose Level B, 40 mg/kg loading dose followed by 20 mg/kg for 1 dose), OR<br>• Enroll 4 subjects at any of the 2 lower dose levels (Dose Level C, 10 mg/kg loading	

**Protocol number:** GMI-1070-103	**Study drug:** Intravenous GMI-1070
dose followed by 5 mg/kg for 1 dose; or Dose Level D, 5 mg/kg loading dose followed by 2.5 mg/kg for 1 dose).<br><br>Safety, PK, and blood flow data will be reviewed after completion of each group of 4 subjects at each site. At that time, a decision will be made to do 1 of the following:<br>• Enroll 4 more subjects at the current dose level, OR<br>• Evaluate titration options and enroll 4 subjects at any other protocol-described dose level.<br><br>This process will continue until a total of 20 subjects have been treated with GMI-1070.<br><br>Note: If enrollment at a site begins after other sites have enrolled in the study, the safety, PK, and blood flow information gained by early-enrolling site(s) may be used to modify dosing by later-enrolling site(s).<br><br>After Dose Level A has been administered to at least 4 subjects at one site, and this site's investigator and the Medical Monitor have determined it appropriate to proceed with another dose level as described above, a new site joining the study may proceed with either of the confirmed dose levels (Dose Level A, or the subsequent chosen dose level) if agreed by the new site's investigator and Medical Monitor.	
**Number of subjects:**<br>The study plans to enroll 4 subjects at a time, up to a total of 20 subjects. An amendment will be considered to increase the total number of subjects if the data from the first 20 subjects support such an increase.<br><br>For each group of 4 subjects at each site, the investigator and Medical Monitor will review the available safety, PK, and blood flow data through 48 ± 12 hours after administration of the first dose of IV GMI-1070. In order to proceed with the study, a consensus must be reached regarding the safety of doing so, as well as whether to proceed to 1 of the alternate dose levels. Any individual site safety concerns discussed will be shared among all participating sites.<br><br>Based on the data from these initial 20 subjects, and if appropriate from a safety perspective, an amendment to the current protocol may be submitted to enroll additional subjects (beyond 20).	
**Study Evaluations:**<br>Subjects will be evaluated in the clinic for up to 11 hours after administration of the first dose. Outpatient follow-up visits to the clinic will take place at 24 ± 3 and 48 ±12 hours post first dose, and on Day 7 ± 2 days. A telephone contact will be made at Day 28 ± 3	

**Protocol number:** GMI-1070-103	**Study drug:** Intravenous GMI-1070
days.<br><br>Plasma sampling for PK and intravital microscopy (IVM) testing for microvascular blood flow, will be performed at the following times:<br>• Predose (on the day of dosing), and<br>• 30 ± 10 minutes, and<br>• 2 ± 0.25, 4 ± 1, 8 ± 2, 24 ± 3, and 48 ± 12 (PK only) hours after administration of the first dose of IV GMI-1070.<br><br>Similarly, plasma sampling for research purposes (biomarkers for adhesion; plasma may be frozen for later research use related to this study) will be performed at the following times:<br>• Predose, and<br>• 4 ± 1, 8 ± 2, 24 ± 3, and 48 ± 12 hours after administration of the first dose of IV GMI-1070.<br><br>Urine sampling for PK will be done over a 6-hour period starting at the beginning of the first dose.	
**Diagnosis and main criteria for admission:**<br>***Inclusion criteria:***<br>To be eligible for inclusion, each subject must fulfill each of the following criteria at screening, and must continue to fulfill these criteria at baseline (if reassessed):<br>1. Age 18 to 50 years<br>2. Established diagnosis of SCD-SS or SCD-Sβ^0-thal<br>3. Subject is at medical baseline, with no evidence of worsening of disease over the last 3 months (as determined by the investigator)<br>4. Expectation that the subject will be available and agree to return for follow-up visits for the full duration of the study<br>5. Ability to cooperate with IVM<br>6. Documented and observed written informed consent<br><br>***Exclusion criteria:***<br>1. Weight of ≥ 100 kg at screening<br>2. Serum creatinine > 1.5 mg/dL<br>3. Alanine transaminase (ALT, also known as SGPT) >2x upper limit of normal (based on clinic laboratory normal range)<br>4. Hemoglobin ≤ 6 g/dL<br>5. Vaso-occlusive crisis (VOC), defined as an episode requiring a visit to a medical facility resulting in medical treatment for pain, within the past 14 days<br>6. Recent (within the past 30 days) major surgery, hospitalization, documented	

**Protocol number:** GMI-1070-103	**Study drug:** Intravenous GMI-1070
bacterial infection requiring antibiotic treatment, or significant bleeding<br>7. Infection or inflammation at the site of the IVM probe placement as determined by the investigator<br>8. Recent (within the past 90 days) cerebrovascular accident or seizure<br>9. Recent (within the past 60 days) transfusion of any type<br>10. Enrollment in a hypertransfusion program<br>11. Currently taking systemic steroids; use of inhaled steroids is acceptable. Use of hydroxyurea or non-steroidal anti-inflammatory drugs (NSAIDs) is acceptable.<br>12. Currently receiving, or has received within the previous 4 weeks, any other investigational agent<br>13. Pregnant or lactating female; or female of childbearing age unable or unwilling to comply with birth control or abstinence during the course of the study<br>14. Medical or psychiatric condition that, in the opinion of the investigator, may pose a risk to the subject for participation or interfere with the conduct or results of the study<br>15. Subjects with a history of alcohol or other substance abuse within the last year	
**Test product, dose, and mode of administration:**<br>GMI-1070 is supplied as sterile solution, 30 mg/mL, in a vial for IV infusion. The test product will be administered using 1 of the following dose regimens (2 doses total, administered at 0 and 10 ± 1 hours):<br>• Dose Level A, 20 mg/kg load followed by 10 mg/kg at 10 ± 1 hours<br>• Dose Level B, 40 mg/kg load followed by 20 mg/kg at 10 ± 1 hours<br>• Dose Level C, 10 mg/kg load followed by 5 mg/kg at 10 ± 1 hours<br>• Dose Level D, 5 mg/kg load followed by 2.5 mg/kg at 10 ± 1 hours	
**Duration of treatment:**<br>• Planned duration of treatment period: 10 ± 1 hours<br>• Planned duration of enrollment and follow-up: 28 ± 3 days from first dose	
**Criteria for evaluation:**<br>***Safety:***<br>• Vital signs, physical examinations, local inspection of the infusion site<br>• Adverse event (AE) reporting<br>• Clinical laboratory testing for systemic safety parameters, including liver function and bilirubin levels, renal function, hematologic testing, and urinalysis<br>***Clinical effect:***<br>• IVM to measure microvascular blood flow<br>• Biomarkers of adhesion, inflammation, and downstream selectin effect in the blood<br>***PK:***<br>• Plasma levels of GMI-1070	

**Protocol number:** GMI-1070-103	**Study drug:** Intravenous GMI-1070
• Urinary excretion of GMI-1070	
**Statistical methods:**<br>Due to the limited population number, descriptive statistics will primarily be used to analyze the study data. Any parameters with noticeable differences will be evaluated using an appropriate method to determine statistical significance. Due to the limited number of plasma concentrations, PK parameters will be estimated by fitting a compartmental model derived from richer datasets in healthy volunteers.	

STUDY SCHEDULE

Table 1: Schedule of Assessments

			IV GMI-1070 Dosing			Post Dosing			
	Screening within 30 days	Pre-Dose Baseline	0 hrs	30±10 min, 2±0.25 hrs, 4±1 hrs, 8±2 hrs	10±1 hrs	24±3 hrs	48±12 hrs		
**Visit**	**Before Day 1**	**Day 1**	**Day 1**			**Day 2**	**Day 3**	**Day 7±2**	**Day 28±3[1]**
Informed consent	✔								
Inclusion/exclusion criteria	✔								
Assignment to dose level		✔							
Demographics	✔								
Medical history	✔	✔							
Physical examination	✔ [2]	✔ [3]				✔ [3]	✔ [3]	✔ [2]	
Height, weight	✔	✔							
Vital signs	✔	✔		✔		✔	✔	✔	
Electrocardiogram	✔							✔	
Biochemistry and hematology[4]	✔	✔		✔ [5]		✔	✔	✔	
High sensitivity C-reactive protein (hsCRP)		✔				✔	✔	✔	
Pregnancy test (urine or serum)	✔	✔							
Urinalysis	✔	✔				✔	✔	✔	
Urine collection over 6 hours for PK				✔					
Biomarkers of adhesion sampling		✔		✔ [6]		✔	✔		
Plasma GMI-1070 PK sampling		✔		✔		✔	✔		
IVM		✔		✔		✔			
GMI-1070 administration			✔		✔				
AEs		✔	✔	✔	✔	✔	✔	✔	✔
Concomitant medication	✔	✔		✔	✔	✔	✔	✔	✔
Telephone contact									✔

[1] Telephone call only is acceptable on Day 28 ± 3. Subject should be queried concerning a positive pregnancy test since last seen.

[2] Full physical examination.

[3] Targeted physical examination.

[4] Complete blood count with differential, platelets, reticulocyte count, alanine transaminase, blood urea nitrogen, creatinine, electrolytes with glucose, lactate dehydrogenase (LDH), bilirubin.

[5] Performed only at 8 hours – hematology (complete blood count) only.

[6] Performed only at 4 and 8 hours.

1 BACKGROUND INFORMATION

This is the third trial for the clinical development of intravenous (IV) GMI-1070. The trial will be conducted in compliance with the protocol, Good Clinical Practice (GCP), and applicable local and federal regulatory requirements. The subject population will comprise adults with stable sickle cell disease (SCD).

GMI-1070 is a pan-selectin antagonist, a compound found to inhibit selectin binding *in vitro* and to inhibit selectin-mediated effects *in vivo*. Selectin binding is a key early step in the inflammatory process leading to leukocyte adhesion and recruitment to inflamed tissue. Selectin binding has been shown to be involved in many disease processes that involve inflammation. There are no other known approved therapeutic agents in this class. This investigational product is being developed for the treatment of vaso-occlusive crisis (VOC) in SCD.

1.1 Sickle Cell Disease

SCD is one of the most prevalent genetic disorders in the United States (US), affecting over 80,000 people.[1] It is a chronic condition with substantial morbidity and mortality, and is responsible for more than 75,000 hospitalizations per year in the US with an average stay of 6.1 days.[2] Both children and adults are affected, and greater mortality is seen in those with more severe disease. The gene is found most commonly in people of African or Arab-Indian descent, and the disease is seen most commonly in these regions or in areas to which these populations have migrated, including the US and Europe.

SCD refers to a group of autosomal recessive inherited disorders of the β-globin gene. A single nucleotide substitution results in the presence of valine instead of glutamic acid in the β-globin chain. The resulting polymerization of hemoglobin S (Hb S) when deoxygenated is the primary indispensable event in the molecular pathogenesis of SCD.[3] Individuals homozygous for Hb S have sickle cell anemia (SCD-SS). Those who are compound (double) heterozygotes have 1 copy of Hb S and 1 copy of either Hb C (SCD-SC), Hb β^{+}-thalassemia (SCD-Sβ^{+}-thal), or Hb β^{0}-thalassemia (SCD-Sβ^{0}-thal).[4]

1.2 Vascular Occlusion in the Pathogenesis of Sickle Cell Complications

SCD is associated with a number of serious and potentially disabling conditions that have similar symptoms but vary in severity by genotype. The most notable complication of SCD is VOC, an extremely painful and serious consequence of SCD presumably resulting from acute ischemic tissue injury. Individuals with the SCD-SS and SCD-Sβ^{0}-thal genotypes tend to experience similar disease severity, which is more severe than for other genotypes. SCD-SC and SCD-Sβ^{+}-thal are associated with a milder form of the disease.[3,4,5]

Over the course of a year, about 60% of patients with SCD-SS will have at least 1 severe VOC, which typically presents as episodes of pain and inflammation at 1 or more sites, of varying degrees of severity, and occurring at varying intervals throughout life. These pain crises, as VOC episodes are also known, are the clinical hallmark of SCD, and are responsible for the vast majority of hospitalizations (> 90%), and result in significant morbidity, mortality, and interruption of daily functioning. The onset of pain is unpredictable, and has been described as periodic, self-limited episodes of excruciating musculoskeletal pain (although visceral, soft tissue, and other locations have been reported), occurring as early as 6 months of age.[6] Other problems include ischemic and hemorrhagic stroke, acute chest syndrome, splenic sequestration, dactylitis, osteonecrosis, priapism, leg ulcers, and nephropathy.[3,7,8,9] Most SCD-related deaths occur during acute VOC, and are due to acute chest syndrome or stroke.[10] Patients can become symptomatic with pain, infection, or splenic sequestration as early as 6 months of age; thus, VOCs are an important cause of morbidity and mortality throughout life, resulting in disruption of the individual's education, psychosocial development, and employment as well as causing severe pain, hospitalization, and premature death.

The etiology of VOC involves dual mechanisms: a mechanical component, by which the sickled red blood cells (RBCs) become caught in the post-capillary venules, and an associated inflammatory response in which white blood cells (WBC) adhere to the endothelium (Figure 1).

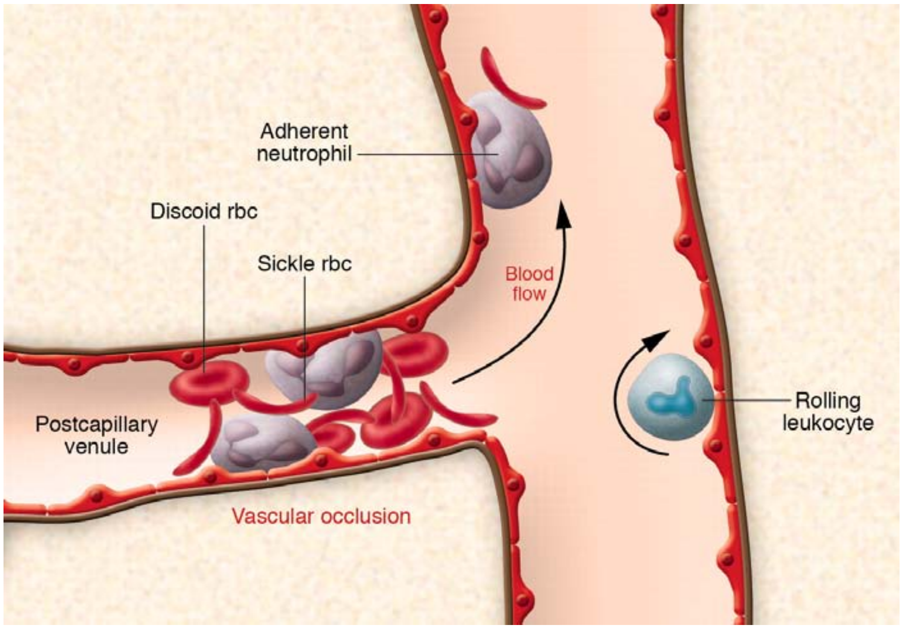

Figure 1: Sickled Red Blood Cells and Neutrophils Occlude Vasculature[4]

The underlying pathogenesis of RBC sickling is the deoxygenation of Hb S, which results in polymerization of the hemoglobin, distortion of the RBC, and loss of deformability of the cell.[11] These "sickled" RBCs are a primary component of the occlusive process and cause vascular injury through interactions with endothelial cells.[7]

The other primary component is adhesion, since the stickiness of the cells causes cell aggregates of WBC-RBC, WBC-WBC, and WBC-platelet to form. This adhesion is selectin-mediated and contributes to the VOC process. Cell aggregates block the vasculature and create a trap for the sickled RBCs, resulting in obstruction of blood flow.[4,12]

Finally, vaso-occlusion resulting from sickled and adherent RBCs also causes slowing of blood flow in post-capillary venules, local tissue hypoxia, and further tissue inflammation, resulting in more deoxygenation and sickling of RBCs, and propagation of the occlusion. This is sometimes called secondary recruitment of sickled cells and occluded vessels.[3] Diapedesis (leukocyte migration into the tissues) and sickle-related microvascular occlusion also takes place in postcapillary venules. Pain is the result of initial and ongoing occlusion and ischemia and is thought to be nociceptive.[1,13,9] It is particularly severe for patients with the SCD-SS genotype, who have been observed to have a higher mortality than other genotypes.[10]

[1] Nociceptive pain is initiated by nociceptors, the nerves which sense and respond to damage (fractures, burns, bumps, bruises, and inflammation) in a particular part of the body. This differs from neuropathic pain, which is the result of an injury or malfunction in the peripheral or central nervous system (CNS).

Of note is that soluble E-selectin, vascular cell adhesion molecules-1 (VCAM-1), and intracellular adhesion molecules-1 (ICAM-1) levels are higher in sickle cell patients at baseline than in normal volunteers. Further evidence suggests that soluble E-selectin and ICAM-1 are increased during acute VOC when compared to baseline in patients with SCD.[14] In addition, increased soluble E-selectin levels have been shown to correlate with increased mortality in SCD patients.[15] Leukocytes adherent to the endothelial cell wall in postcapillary and collecting venules interact with sickled RBCs, leading to vaso-occlusion in sickle cell mice. However, sickle cell mice lacking E- and P-selectin were protected from developing vaso-occlusion.[16] An understanding of the role that selectins play in cell aggregation and the pathophysiology of VOC is increasing.[3,17]

1.3 Intravital Microscopy

Intravital microscopy (IVM) is a non-invasive imaging technique used for studying dynamic cell interactions and microvascular morphometry in living tissue. Variations of this technique have been used to measure microvascular blood flow and perfusion in both human and animal studies for multiple diseases, including diabetes, cardiovascular disease, and more recently SCD. The methods used in humans are non-invasive and involve application of a probe to an accessible vascular tissue such as the bulbar conjunctiva or sublingual mucosa. In this manner, blood vessels can be directly observed using optical or Doppler techniques. Measurements such as cell movement, vessel morphometry, and vessel size are taken, and calculations made of microvascular blood flow and perfusion. Numerous groups have used such techniques to evaluate blood flow in various disease models in animals[18,19,20] as well as in humans.[21,22] A few have used the technique to evaluate blood flow in sickle cell patients, at baseline and in VOC, as well.[23,24,25,26] Limited work has been done to evaluate changes in microvascular blood flow resulting from direct intervention, including 1 study in patients experiencing VOC who had improvement in microvascular blood flow resulting from treatment with an anti-adhesive agent (Poloxamer-188).[25] Thus, the use of this non-invasive technique to document such responses to treatment in the context of SCD is promising.

1.4 Treatment of Vaso-Occlusive Crisis

Nonpharmacologic management of pain includes cutaneous stimulation (transcutaneous electrical nerve stimulation), heat, cold, vibration, distraction, relaxation, massage, music, guided imagery, self-hypnosis, self-motivation, acupuncture, and biofeedback.[27]

Pharmacologic management of pain includes the use of 3 major classes of compounds: non-opioids (nonsteroidal anti-inflammatory drugs [NSAIDs], topical agents, tramadol, and corticosteroids), opioids, and adjuvants

(antihistamines, antidepressants, benzodiazepines, and anticonvulsants).[27] Adjuvant treatments for pain are typically used for chronic pain. NSAIDs are the first line of therapy for acute VOC pain, and are typically prescribed on an outpatient basis. Opioids in repeated and increasing doses are the first line of treatment for emergency department or inpatient care required for acute VOC. Common drugs include codeine (oral, for mild pain); or for moderate to severe pain, IV morphine, or morphine-equivalent opioids such as oxymorphone, levophanol, meperidine, fentanyl, and methadone.[27] Opioid agonists may also be used in the management of sickle cell pain in adults. They decrease or modify the perception of pain at the level of the CNS.[27]

Other supportive measures for VOC include hydration, transfusion, and treatment of any concurrent infections. No treatment is available to interfere with the sickling or vaso-occlusive process.

Current long-term medical management of SCD includes use of hydroxyurea, which is used to increase Hb F concentration and reduce the number of pain crises by preventing their development.[27]

1.5 Nonclinical Information

A summary of nonclinical studies is provided in the following sections. Additional information may be found in the Investigator’s Brochure.

1.5.1 Pharmacology

In vitro and *in vivo* pharmacodynamic studies demonstrate that GMI-1070 administration can inhibit selectin binding and biological activity as it relates to induction of selectin-mediated inflammation. *In vitro* studies have demonstrated that GMI-1070 blocked binding of all 3 selectins (E-, P-, and L-selectin), and inhibited the selectin-mediated leukocyte rolling and adhesion.

In vivo studies in a mouse model for SCD showed that treatment with 20 mg/kg of GMI-1070 increased blood flow, decreased adhesion of cells, and increased survival. In a delayed-type hypersensitivity model, treatment with GMI-1070 (10 and 20 mg/kg) inhibited T-cell migration to the site of inflammation. Administration of GMI-1070 (20 mg/kg IV) in the diabetic mouse model inhibited the inherent selectin-mediated increased rolling and adhesion of leukocytes. In an acute myocardial injury rate model, the infarct size is the result of a selectin-dependent inflammatory response. Administration of GMI-1070 (10 or 20 mg/kg IV) in this model resulted in a decrease in infarct size.

A CNS safety pharmacology study was conducted in CD-1 mice. No treatment-related effects were observed in the functional observational battery of tests when the mice were administered IV GMI-1070 (37.5, 150, or 300 mg/kg).

Results of 2 human ether-a-go-go-related gene assays with GMI-1070 (0.5, 1.0, 5.0, and 10.0 mg/mL for the first study; 0.5, 1.0, 3.0, 7.0, and 10.0 mg/mL for the second study) gave no evidence that GMI-1070 administration to humans would have any untoward effect on the cardiovascular system in regard to QT prolongation. These study results are consistent with the results of the 14-day repeat-dose toxicology study in cynomolgus monkeys noted below, in which IV administration of GMI-1070 (up to 75 mg/kg) had no apparent effect on QT intervals as determined by electrocardiogram (ECG).

1.5.2 Pharmacokinetics

A single IV dose of GMI-1070 administered to mice (20 mg/kg), rats (20 mg/kg), and monkeys (150 mg/kg), showed rapid systemic exposure with a consistent time to maximum concentration (t_{max}) of 0.08 hours for all 3 species. Maximum concentration (C_{max}) was slightly lower in mice than rats (107.83 and 153.0 µg/mL, respectively). The area under the curve ($AUC_{(0-\infty)}$) was higher in rats (233.45 hours µg/mL) than mice (107.12 hours µg/mL), but the half-life ($t_{1/2}$) was similar for mice, rats, and monkeys at 1.25 hours, 1.4 hours, and <3 hours, respectively.

1.5.3 Toxicology

Acute (single dose) toxicity testing of GMI-1070 in mice (3, 10, 30, 100, or 300 mg/kg) and cynomolgus monkeys (18.75, 37.5, 75, and 150 mg/kg) showed no treatment-related effects. Therefore, no maximum tolerated dose was determined. Results of the 14-day repeat-dose IV toxicology study in mice indicated a no –observed –adverse effect level (NOAEL) of the highest dose tested, 150 mg/kg. The results of the 14-day repeat-dose IV toxicology study in cynomolgus monkeys showed a NOAEL of the highest dose tested, 75 mg/kg.

Results of the standard battery of genetic toxicology studies indicated that GMI-1070 is not mutagenic or genotoxic. In a local tolerance study of GMI-1070 in New Zealand White rabbits, GMI-1070 was administered by IV injection in the right marginal ear vein (300 mg/animal) and by perivascular injection adjacent to the left marginal ear vein (3 mg/kg). No evidence of treatment-related local injection site toxicity or irritation was observed.

1.6 Clinical Information

A summary of clinical information is provided in the following sections. Additional information may be found in the Investigator’s Brochure.

1.6.1 Safety

Study GMI-1070-101 evaluated single doses of IV GMI-1070 at 2, 5, 10, 20, and 40 mg/kg. Study GMI-1070-102 evaluated multiple doses of 5, 10, and 20 mg/kg for 4 days, and a loading dose of 40 mg/kg followed by multiple doses of 20 mg/kg for 2 days. These studies were evaluated on a blinded basis in 72 healthy adult subjects (54 active, 18 placebo) as of 2 February 2009. GMI-1070 has demonstrated an excellent safety and tolerability profile after both single and multiple ascending doses as demonstrated by:

- No clinically significant ECG or physical exam findings
- All adverse events (AEs) were Grades 1 or 2
- No serious AEs (SAEs) occurred

Mild local irritation at the site of IV administration was observed to occur more frequently in higher dose groups with multiple doses. Two subjects in the high dose group of these Phase 1 studies have developed a rash. One subject is known to have received a dose of GMI-1070, 40 mg/kg. Assignment (to GMI-1070 or placebo) is still blinded for the second subject, but itching and spread of the rash required termination of dosing after 3 doses and treatment for itching. See Investigator's Brochure for details. None of these observations prevented dose escalation or progression to the next study.

It was therefore concluded by the investigator and the Medical Monitor that all doses observed in both studies demonstrated minimal safety concerns for continuing clinical trials at the tested dose levels, up to and including multiple doses of 20 mg/kg for 4 days and alternatively a loading dose of 40 mg/kg followed by 20 mg/kg for 2 days.

1.6.2 Pharmacokinetics

In the single-dose study (GMI-1070-101), pharmacokinetics (PK) data showed detectable levels of drug, consistent PK parameters among subjects, and linear kinetics between groups. Plasma concentrations (C_{max}) and $AUC_{(0-t)}$ showed linear increases from the 2 mg/kg to the 40 mg/kg dose. Clearance (CL), renal clearance (CL_r), apparent volume of distribution at the terminal phase (Vz), and $t_{½}$ did not change with dose. The mean value for CL_r ranged from 15.1 to 20.5 mL/minute, with an overall mean of 18.3 mL/min. CL_r was not dependent on plasma concentration. Over 90% of GMI-1070 was excreted in the urine.

In the multiple-dose study (GMI-1070-102), PK data are available for 3 dose levels: 5, 10, and 20 mg/kg. Key parameters on Days 1 and 5 for these groups (CL, Vz, and $t_{1/2}$) are consistent with those from the comparable dose level in Study GMI-1070-101.

Figure 2 below shows a semi-log graph of the single dose PK parameters from IV GMI-1070 at a range of doses from 2-40 mg/kg (GMI-1070-101 study).

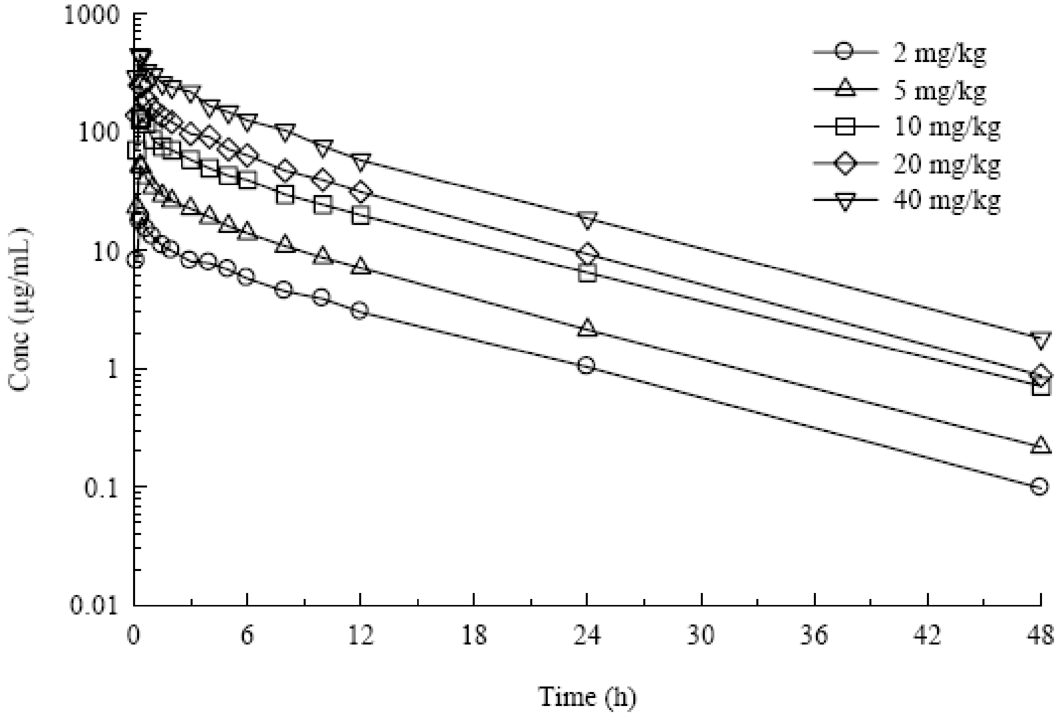

Figure 2: IV GMI-1070 Pharmacokinetics with Single Dose in Volunteers: 2-40 mg/kg

2 STUDY OBJECTIVES AND PURPOSE

2.1 Rationale for this Study

GMI-1070 is a potent, rationally designed glycomimetic inhibitor of E-, P-, and L-selectins *in vitro*. GMI-1070 has been shown to inhibit inflammation in several animal models of disease, including the VOC of SCD. This investigational product is being developed for the treatment of VOC in SCD. In the present study, adult SCD patients who are not in VOC will be enrolled. In future clinical studies (not discussed here), IV GMI-1070 will be evaluated for safety and effect in sickle cell patients who are experiencing VOC.

Safety and PK data collected in the 2 prior studies of IV GMI-1070 in healthy volunteers have demonstrated an excellent safety profile and linear, predictable PK parameters at a range of doses for up to 4 days of treatment. The present

study seeks to build on this data by evaluating the safety, PK, and correlation with microvascular effect of 1 day of dosing with IV GMI-1070 in adult sickle cell patients who are at their stable medical baseline, not in VOC. The initial dose level being tested in this study is in the mid-range of those studied in the previous human studies, and subjects will receive 2 doses (1 day) of IV GMI-1070 as compared to 13 doses (4 days) in the previous multiple dose study. The starting dose level in this study, therefore, is substantially lower exposure to the study drug than in previous human studies. This data thus gives a solid platform to launch this current study as a reasonable first evaluation of IV GMI-1070 in sickle cell patients.

The rationale for the present study is to explore:

- Safety in the SCD population, and
- PK/pharmacodynamic relationships by correlation of blood levels of GMI-1070 with the effect on microvascular blood flow parameters in adults with SCD.

2.2 Study Objectives

2.2.1 Primary

- Evaluate the safety of multiple IV doses of GMI-1070 in adults with SCD.

2.2.2 Secondary

- Evaluate the PK of multiple IV doses of GMI-1070 in adults with SCD.
- Evaluate the microvascular blood flow before and after treatment with IV GMI-1070 in adults with SCD.
- Evaluate biomarkers of adhesion, inflammation, and downstream selectin effect in the blood before and after treatment with IV GMI-1070 in adults with SCD.

3 STUDY DESIGN

This will be an open-label, dose titration study of IV GMI-1070 in adults with stable SCD. Subjects will receive IV GMI-1070 at dose levels based on findings in earlier human studies. Study drug will be administered in the Clinical Research Center (CRC), day hospital, or equivalent clinical setting.

Titration for this study may be done either up or down and not necessarily in a step-wise fashion. At the beginning of the study, 4 subjects at each site will receive Dose Level A, which consists of a 20 mg/kg loading dose of IV GMI-1070 followed by a single 10 mg/kg dose of IV GMI-1070. Thus, subjects will

receive a total of 2 doses: 1 loading dose (20 mg/kg) in the morning at 0 hours, and 1 single dose (10 mg/kg) in the evening at 10 ± 1 hours.

After these first 4 subjects at each site have been treated, safety, PK, and blood flow data from this first group will be reviewed by the investigator and the Medical Monitor. After this review, a site-specific decision will be made to do one of the following:

- Enroll 4 more subjects at this dose level (continue at Dose Level A); OR
- Enroll 4 subjects at the higher dose level (Dose Level B, 40 mg/kg loading dose followed by 20 mg/kg for 1 dose); OR
- Enroll 4 subjects at any of the 2 lower dose levels (Dose Level C, 10 mg/kg loading dose followed by 5 mg/kg for 1 dose; or Dose Level D, 5 mg/kg loading dose followed by 2.5 mg/kg for 1 dose).

Safety, PK, and blood flow data will be reviewed after completion of each group of 4 subjects at each site. At that time a decision will be made to do one of the following:

- Enroll 4 more subjects at the current dose level; OR
- Evaluate titration options and enroll 4 subjects at any other protocol-described dose level.

This process will continue until a total of 20 subjects have been treated with GMI-1070. Refer to Figure 3 in Section 3, Study Titration Flow, for a diagram of study drug dosing at each site.

Note: If enrollment at a site begins after other sites have enrolled in the study, the safety, PK, and blood flow information gained by early-enrolling site(s) may be used to modify dosing by later-enrolling site(s).

After Dose Level A has been administered to at least 4 subjects at one site, and this site’s investigator and the Medical Monitor have determined it appropriate to proceed with another dose level as described above, a new site joining the study may proceed with either of the confirmed dose levels (Dose Level A, or the subsequent chosen dose level) if agreed by the new site’s investigator and Medical Monitor.

Subjects will be evaluated in the clinic for up to 11 hours after administration of the first dose. Outpatient follow-up visits to the clinic will take place at 24 ± 3 and 48 ±12 hours post first dose, and on Day 7 ± 2 days. A telephone contact will be made at Day 28 ± 3 days.

Plasma sampling for PK and IVM testing for microvascular blood flow will be performed at the following times:

- Predose (on the day of dosing), and
- 30 ± 10 minutes, and
- 2 ± 0.25, 4 ± 1, 8 ± 2, 24 ± 3, and 48 ± 12 (PK only) hours after administration of the first dose of IV GMI-1070

Similarly, plasma sampling for research purposes (biomarkers for adhesion; plasma may be frozen for later research use related to this study) will be performed at the following times:

- Predose, and
- 4 ± 1, 8 ± 2, 24 ± 3, and 48 ± 12 hours after administration of the first dose of IV GMI-1070

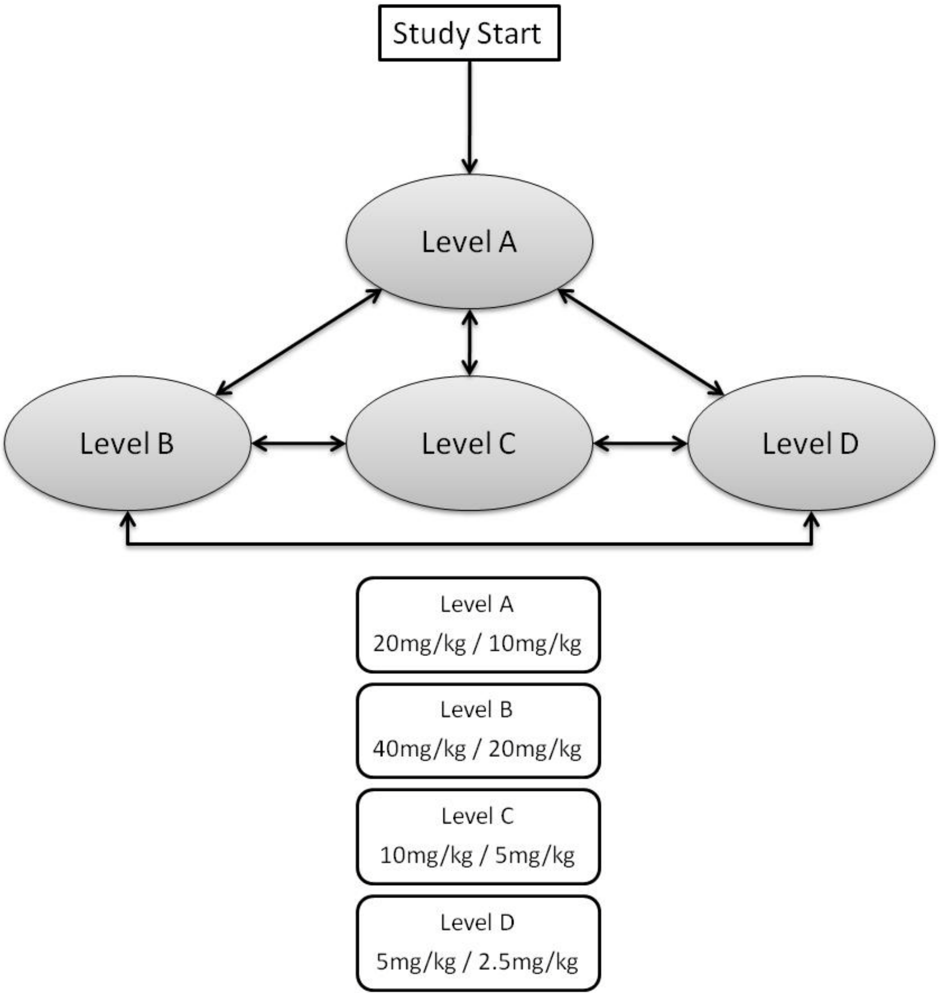

Figure 3: Study Titration Flow Chart

4 SELECTION AND WITHDRAWAL OF SUBJECTS

4.1 Number of Subjects

The study plans to enroll 4 subjects at a time, up to a total of 20 subjects. An amendment will be considered to increase the total number of subjects if the data from the first 20 subjects support such an increase.

4.2 Inclusion Criteria

To be eligible for inclusion, each subject must fulfill each of the following criteria at screening, and must continue to fulfill these criteria at baseline (if reassessed):

1. Age 18 to 50 years
2. Established diagnosis of SCD-SS or SCD-Sβ^0-thal
3. Subject is at medical baseline, with no evidence of worsening of disease over the last 3 months (as determined by the investigator)
4. Expectation that the subject will be available and agree to return for follow-up visits for the full duration of the study
5. Ability to cooperate with IVM
6. Documented and observed written informed consent

4.3 Exclusion Criteria

Subjects will be excluded from the study if any of the following criteria are met at screening or at baseline (if reassessed):

1. Weight of ≥ 100 kg at screening
2. Serum creatinine > 1.5 mg/dL
3. Alanine transaminase (ALT/SGPT) > 2x upper limit of normal (based on clinic lab normal range)
4. Hemoglobin ≤ 6 g/dL
5. VOC, defined as an episode requiring a visit to a medical facility resulting in medical treatment for pain, within the past 14 days
6. Recent (within the past 30 days) major surgery, hospitalization, documented bacterial infection requiring antibiotic treatment, or significant bleeding
7. Infection or inflammation at the site of probe placement as determined by investigator
8. Recent (within the past 90 days) cerebrovascular accident or seizure
9. Recent (within the past 60 days) transfusion of any type
10. Enrollment in a hypertransfusion program
11. Currently taking systemic steroids; use of inhaled steroids is acceptable. Use of hydroxyurea or NSAIDs is acceptable

12. Currently receiving, or has received within the previous 4 weeks, any other investigational agent
13. Pregnant or lactating female; or female of childbearing age unable or unwilling to comply with birth control or abstinence during the course of the study
14. Medical or psychiatric condition that, in the opinion of the investigator, may pose a risk to the subject for participation or interfere with the conduct or results of the study
15. Subjects with a history of alcohol or other substance abuse within the last year

4.4 Withdrawal of Subjects

A subject is free to withdraw from the study at any time for any reason without prejudice to their future medical care by the physician or at the institution. The investigator or Sponsor may also withdraw the subject at any time in the interest of subject safety. The primary reason for withdrawal must be recorded on the withdrawal form in the case report form (CRF). If a subject is withdrawn for more than 1 reason, each reason should be documented and the most medically significant reason should be noted on the CRF.

The withdrawal of a subject from the study should be discussed where possible with the Medical Monitor before the subject stops investigational product. If investigational product is discontinued, the final evaluations will be performed as completely as possible. Subjects who drop out of the study prior to obtaining endpoint data from hour 24 will be replaced by the next available subject. Any comments (spontaneous or elicited) or complaints made by the subject and the reason for termination, date of stopping investigational product, and the total amount of investigational product taken must be recorded in the CRF.

The Sponsor reserves the right to terminate the study at any time.

Reasons for withdrawal may include:

- If consent is withdrawn or the subject refuses to continue treatment and/or procedures/observations
- If the clinical condition under study requires alternative treatment
- Occurrence of unmanageable AEs or if the subject requires concomitant medication disallowed under this protocol (see Section 5.2)
- If the subject becomes pregnant
- For other reasons (eg, significant protocol violation or non-compliance)

At least 3 documented attempts must be made to contact any subject lost to follow-up, 1 of which must include sending a certified letter to the subject’s last

known address, requesting that they return to the study site for final safety evaluations.

The Sponsor may be contacted if clarification is required on a case-by-case basis.

5 STUDY AND CONCOMITANT TREATMENTS

5.1 GMI-1070

The official product name, as indicated in the Investigator's Brochure, is 'GMI-1070 Injection, 30 mg/mL.' However, in order to allay any confusion regarding the route of administration in this protocol, the product has been referred to as 'GMI-1070.' For further details regarding the exact composition of the product, please refer to the Investigator's Brochure.

Investigational product will be supplied in vials, each containing 250 mg of liquid formulation of GMI-1070 at a 30 mg/mL concentration. Each study drug administration will occur over a period of 20 minutes from the start of the IV infusion, and will be followed by a saline flush.

The test product will be administered using 1 of the following dose regimens (2 doses total, administered at 0 and 10 ± 1 hours):

- Dose Level A, 20 mg/kg load followed by 10 mg/kg at 10 ± 1 hours
- Dose Level B, 40 mg/kg load followed by 20 mg/kg at 10 ± 1 hours
- Dose Level C, 10 mg/kg load followed by 5 mg/kg at 10 ± 1 hours
- Dose Level D, 5 mg/kg load followed by 2.5 mg/kg at 10 ± 1 hours

A written authorization (prescription/order/instruction) signed by an appropriately licensed physician, in this case the investigator, is required prior to providing any subject with investigational product.

Note: If enrollment at a site begins after other sites have enrolled in the study, the safety, PK, and blood flow information gained by early-enrolling site(s) may be used to modify dosing by later-enrolling site(s).

After Dose Level A has been administered to at least 4 subjects at one site, and this site's investigator and the Medical Monitor have determined it appropriate to proceed with another dose level as described above, a new site joining the study may proceed with either of the confirmed dose levels (Dose Level A, or the subsequent chosen dose level) if agreed by the new site's investigator and Medical Monitor.

5.1.1 Allocation of Subjects to Treatment

This is an open-label study in which subjects will be enrolled into dose levels on a first-come, first-served basis. The study will not be blinded or randomized.

5.1.2 Labeling, Packaging, Storage, and Handling

The investigational product will be shipped to a designee at the study site and must be stored in a pharmacy or locked and secured in a storage facility with temperature control, accessible only to those individuals authorized by the investigator or designee.

The Sponsor will supply sufficient quantities of IV GMI-1070 to allow completion of this study. The lot numbers, manufacture dates, and expiration dates (if available) of the drugs supplied will be recorded in the final report.

Investigational product will be supplied to the pharmacy in vials, each containing 250 mg of liquid formulation of GMI-1070 at a concentration of 30 mg/mL. Vials will be labeled with the following label:

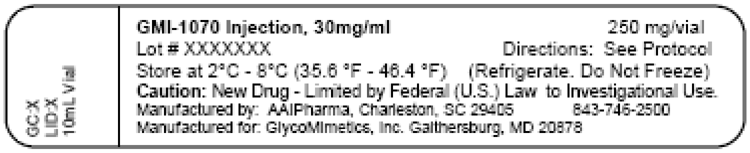

Study drug must be refrigerated at a controlled temperature between 2° and 8°C. Temperature monitoring is required at the storage location to ensure that the investigational product is maintained within this temperature range. The investigator or designee is responsible for ensuring that the temperature is monitored throughout the total duration of the trial and that records are maintained; the temperature should be monitored continuously. Excursion from the established range will require site investigation as to cause and remediation. GMI will determine the ultimate impact of any excursions and will provide supportive documentation.

5.1.3 Compliance and Drug Accountability

Investigational product must be used only as directed in the protocol. The investigator or designee will be provided with forms to enable accurate, written records of all investigational product received from GMI. These records will be kept by the investigator, pharmacist, or designee of either individual.

At the end of the study, and only upon written authorization by GMI, all unused stock will be sent to a designated contractor on behalf of GMI. Investigational

products returned to GMI-designated contractors must be counted and verified by site personnel and GMI (or designated contract research organization [CRO]) prior to shipment. All certificates of delivery/drug receipts, and/or return forms must be signed prior to shipment. Investigational products for return must be packed in a tamper-evident manner to ensure integrity by receiving contractor. All investigational product returned must be in accordance with local, state, and national laws.

Based on entries in the site drug accountability forms, it must be possible to reconcile drug delivered with that used and returned. All investigational product must be accounted for and all discrepancies investigated and documented appropriately.

5.2 Concomitant Medications

All concomitant medications should be recorded at Screening, confirmed predose, and recorded again during the study visits. Subjects currently taking anti-inflammatory agents or steroids will be excluded from enrollment. The use of hydroxyurea and NSAIDs will be allowed.

6 STUDY PROCEDURES

6.1 Study Schedule

Refer to the Schedule of Assessments, Table 1, for a complete list of procedures and times for each assessment.

6.1.1 Screening Visit

The Screening Visit should occur within 30 days prior to initiation of treatment. All prospective subjects will have the study explained in non-technical terms by a member of the research team or a member of the clinic staff. The nature of the drug substance to be evaluated will be explained together with potential hazards involving possible adverse reactions. Written informed consent will be obtained from each subject prior to dosing.

Subjects will have to meet all inclusion/exclusion criteria before being enrolled in the study (see Section 4).

6.1.2 Day of GMI-1070 Administration

6.1.2.1 Baseline

Baseline evaluations will be performed the day of IV GMI-1070 dosing, prior to drug administration. Eligible subjects will be assigned to a specific dose level by the investigator, in accordance with the protocol (refer to Section 5.1.1).

6.1.2.2 Dosing

The weight measured at Screening will be used to determine the appropriate dose administered throughout the study. Subjects will be dosed with IV GMI-1070 for a total of 2 doses over 10 ± 1 hours (as specified by dose level, refer to Section 5.1). GMI-1070 is supplied as sterile solution for infusion. Each study drug administration will occur over a period of 20 minutes from the start of the IV infusion, and will be followed by a saline flush.

The calendar date and 24-hour clock time of the beginning and end of each infusion will be recorded on the CRF.

6.2 Study Evaluations and Procedures

6.2.1 Safety

6.2.1.1 Physical Examination

A full physical examination includes a review of the following body systems:

- General Appearance
- Skin
- Head, Ears, Eyes, Nose, Throat
- Spine/Neck/Thyroid
- Respiratory
- Cardiovascular
- Abdomen (including liver and kidneys)
- Nervous System
- Musculoskeletal

A targeted physical examination includes body systems selected at the discretion of the investigator.

Any abnormalities or changes in intensity noted during the review of body systems should be documented and reported on the appropriate CRF page. If a new clinically significant finding (ie, not noted at screening) occurs at the baseline examination or subsequently, an AE form must be completed. In

addition, resolution of any abnormal findings during the study will be noted in the medical record and/or the CRF if clinically significant.

6.2.1.2 Vital Signs

Complete vital signs are considered to be body temperature, respirations, blood pressure, and pulse (taken after 5 minutes seated).

When the time of vital signs measurement coincides with a blood draw, the vital signs will be taken approximately 10 minutes before the scheduled blood draw.

6.2.1.3 Electrocardiogram

A 12-lead ECG will be performed according to standard procedure at the investigational site.

6.2.1.4 Clinical Laboratory Evaluations

All laboratory assays will be performed locally according to the laboratory's normal procedures. Reference ranges will be supplied by the local laboratory. Lab assays may be repeated once to rule out lab error, before determining if results are normal or abnormal. It is recognized that in SCD lab values that are out of the reference range may in fact be baseline for that individual. When test results are outside the reference range, the investigator will indicate on the CRF whether the result is considered to be clinically significant or not. Out of range results not considered to be clinically significant by the investigator will not be considered abnormal for the purposes of this study. Abnormal laboratory values which are unexpected or not explained by the subject's clinical condition should be repeated until confirmed, explained, or resolved. Data to be collected are noted below.

Table 2: Clinical Laboratory Evaluations

**Hematology**	
Hemoglobin	Platelet count
Hematocrit	WBC count with differential
RBC count	High sensitivity C-reactive protein (hsCRP)
Reticulocyte count	
**Serum Chemistry**	
Blood urea nitrogen	ALT (SGPT)
Creatinine	AST (SGOT)
Electrolytes with glucose	Total and Direct bilirubin
	Lactate dehydrogenase (LDH)

**Urinalysis**	
pH	Bilirubin
Specific gravity	Blood
Protein	Nitrite
Glucose	Urobilinogen
Ketones	Leukocyte esterase
If urinalysis is positive for blood or leukocyte esterase, a microscopic examination for RBC, WBC, bacteria, and casts will be performed.	

6.2.1.5 Pregnancy Test

Female subjects should be either post-menopausal (amenorrhea for at least 12 consecutive months), surgically sterile, or women of child-bearing potential with a negative urine or serum beta human chorionic gonadotropin pregnancy test prior to entering the study and who are using or agree to use at least 1 acceptable method of contraception. Acceptable contraceptives include intrauterine devices, hormonal contraceptives (oral, depot, patch, or injectable) and double barrier methods such as condoms or diaphragms with spermicidal gel or foam.

Women of child-bearing potential must be advised to use acceptable contraceptives throughout the study period and for 30 days after the last dose of investigational product. If hormonal contraceptives are used, they should be taken according to the package insert. Women of child-bearing potential who are not currently sexually active must agree to use acceptable contraception, as defined above, if they decide to become sexually active during the period of the study and for 30 days after the last dose of investigational product.

At the Day 28 ± 3 follow up telephone call, women of child-bearing potential will be asked if they have had a positive pregnancy test since taking place in the study. The response will be documented in the CRF. Any pregnancies will be handled as described in Section 7.1.

6.2.2 Non-Safety Sampling

Plasma sampling for PK, and IVM testing for microvascular blood flow, will be performed at the following times:

- Predose (on the day of dosing), and
- 30 ± 10 minutes, and
- 2 ± 0.25, 4 ± 1, 8 ± 2, and 24 ± 3 hours, and
- 48 ± 12 (PK only) hours after administration of the first dose of IV GMI-1070.

Urine sampling for PK will be done over a 6-hour period starting at the beginning of the first dose.

Plasma sampling for biomarkers will be performed at the following times (samples may be frozen for later research use related to this study):

- Predose, and
- 4 ± 1, 8 ± 2, 24 ± 3, and 48 ± 12 hours after administration of the first dose of IV GMI-1070.

Outpatient follow-up visits to the clinic will take place at 24 ± 3 and 48 ± 12 hours post first dose, and on Day 7 ± 2 and a telephone follow-up at Day 28 ± 3.

6.2.3 Clinical Effect

Specific details required for performance of these study evaluations and procedures may be found in the Study Operations Manual.

- IVM to measure microvascular blood flow will be performed at times specified on the study schedule.
- Biomarkers of adhesion and downstream selectin effect in the blood. Blood samples of 4 mL will be collected via direct venipuncture at times specified on the study schedule. Specific details for sample handling, storage, and shipping may be found in the Study Operations Manual.

6.2.4 Pharmacokinetic Samples

Blood samples of 4 mL each will be collected via direct venipuncture at times specified on the study schedule. Samples will be shipped according to the Sponsor's instructions. Specific details for sample handling, storage, and shipping may be found in the Study Operations Manual.

6.2.5 Review to Determine Next Dose Level

When 4 subjects have been studied (in any level) at the individual site, the data for these subjects will be reviewed by the investigator and Medical Monitor in order to determine the next dose to be administered. The data for the first 48 hours for each subject, as available, will be reviewed.

Table 3: Data Reviewed to Determine Next Dose Level

	**Safety**	**PK**	**Effect**
**Data to review:**	Physical examination, AEs, clinical labs, vital signs	GMI-1070 plasma levels	IVM data
**To determine:**	If dose-limiting toxicity is seen*	If plasma levels are consistent with expected levels	If difference in IVM data is seen pre- and post-dose

*Dose-limiting toxicity is defined in Section 7.4.

Based on review of these data, and determination of the above, dosing may continue as follows using 1 of the dose levels described in this protocol:

- If dose-limiting toxicity is seen, the decision will be made based on safety data.
  - If dose-limiting toxicity is seen, and continued dosing at a lower dose level is believed to be appropriate (as determined by the investigator and Medical Monitor), the study may continue at a lower dose level.
  - If dose-limiting toxicity is seen, and continued dosing at any level is not warranted (as determined by the investigator and Medical Monitor), the study will be halted for further review of the data.

- If no dose-limiting toxicity is seen, the decision will be made based on effect data.
  - If a microvascular blood flow effect is not clearly detectable by IVM, the study may continue at the same dose to gather more data, or increase to a higher dose level.
  - If a microvascular blood flow effect is clearly detectable, the study may continue at the same dose, or decrease to a lower dose level.

Any individual site safety concerns discussed, that could have a more global impact, will be shared among all participating sites.

7 ADVERSE AND SERIOUS ADVERSE EVENTS

7.1 Safety Monitoring

All AEs occurring from enrollment through Day 28 ± 3 will be recorded. An AE is any unintended medical occurrence in a subject or clinical investigation subject administered a pharmaceutical product that does not necessarily have a causal relationship with this treatment (International Conference on Harmonisation/World Health Organization [ICH/WHO]). An AE can, therefore, be any unfavorable and unintended sign (including an abnormal laboratory finding, for example), symptom, or disease temporally associated with the use of a medical product, whether or not considered related to the medical product (ICH/WHO).

The subjects will be instructed to inform the investigator or clinic staff of any AEs and intercurrent illnesses experienced during the trial. The inquiry will be posed in a non-specific manner using open-ended questions so as not to bias the response (eg, How are you today?).

Patients with SCD, as a result of this condition, commonly have abnormal values for hemoglobin, hematocrit, WBC count, bilirubin, aspartate transaminase (AST,

also known as SGOT), LDH, CRP, hematuria, and other laboratory and clinical findings. It is assumed that abnormalities in physical exam or laboratory findings consistent with the subject's underlying diagnosis of SCD will be present prior to dosing and therefore will not be treatment-emergent AEs (TEAEs). However, if changes believed to be related to the diagnosis of SCD occur after the initiation of dosing, the investigator shall note whether they are considered AEs (clinically significant changes) or not clinically significant, and if designated as AEs, the relationship to GMI-1070.

Any subject who has an AE (whether serious or non-serious) will be evaluated by the investigator, or other monitoring physician, and will be treated, referred to the subject's own physician, and/or followed up until the symptoms or values return to normal or acceptable levels, as judged by the investigator. A physician, either at the study clinic or at a nearby hospital emergency room, will administer treatment for any SAEs. Where appropriate, medical tests and examinations will be performed to document resolution of event(s).

Pregnancy occurring during a study in a female study subject will be documented in a note to file and reported as a protocol deviation to the Institutional Review Board (IRB). Pregnancy itself is not an SAE but will be reported as an AE. The investigator or designee will discontinue the pregnant subject from the treatment aspects of the study (PK testing, IVM, and IV GMI-1070 administration), continue to follow her per protocol for safety outcomes only, and advise her to seek prenatal care and counseling from her primary care provider.

The site clinical staff will request the pregnant subject to notify the site of the outcome of the pregnancy (ie, birth, loss, or termination). To help ensure this, the site clinical staff will follow-up with the subject until the end of pregnancy, with the subject's consent. This request and the subject's response will be documented in the subject's source document.

7.2 Reporting

All AEs occurring during this clinical trial will be recorded. The investigator will review each event and assess its relationship to drug treatment (unrelated, remote, possible, probable, or definite). Worsening of pre-treatment events after initiation of investigational product must be recorded as new AEs.

Each sign or symptom reported will be graded in accordance with the WHO Common Toxicity Criteria (Appendix 1) where Grade 1 = mild, Grade 2 = moderate, Grade 3 = severe, Grade 4 = life-threatening or disabling, and Grade 5 = death related to AE. The date of AE onset, time of onset, duration, severity, relationship, outcome, and use of concomitant treatment will be recorded for each event.

For the purposes of this study, VOC shall be defined as an episode requiring a visit to a medical facility resulting in medical treatment for pain. It is recognized that VOC and pain are frequent and expected events in SCD, and the use of narcotics for these episodes is often required and considered routine. When determining grade of an AE for VOC or pain, the investigator shall use the following classification (noted below), rather than the need for narcotics as described in the WHO Common Toxicity Criteria.

If the AE does not have a toxicity grade listed in Appendix 1, the following severity classifications will be used.

- Mild - The AE is easily tolerated and does not interfere with daily activity
- Moderate - The AE interferes with daily activity, but the subject is still able to function
- Severe - The AE is incapacitating and requires medical intervention

The relationship of each AE to study drug will be assessed using the following definitions:

Definite:

- Distinct temporal relationship with drug treatment
- Event cannot be explained by subject's clinical state or other factors

Probable:

- Reasonable temporal relationship with drug treatment
- Event cannot easily be explained by subject's clinical state or other factors

Possible:

- Reasonable temporal relationship with drug treatment
- Event could be explained by subject's clinical state or other factors

Remote:

- Poor temporal relationship with drug treatment
- Event easily explained by subject's clinical state or other factors

Unrelated:

- Event occurring before dosing
- Event or intercurrent illness due wholly to factors other than drug treatment

7.3 Serious Adverse Event

An SAE is any untoward medical occurrence that (by WHO and ICH definitions):

- Results in death
- Is life-threatening
- Results in permanently disabling or incapacitating condition
- Requires inpatient hospitalization
- Prolongs inpatient hospitalization
- Is a congenital anomaly/birth defect
- In addition, is an important medical event that may not result in death, be life-threatening, or require hospitalization but which, based upon appropriate medical judgment, may jeopardize that subject and may require medical or surgical intervention

All SAEs will be reported to the CRO SAE Hotline within 1 working day and followed by written reports within 2 working days, whether or not the SAEs are deemed drug-related.

If an SAE occurs, the following CRO SAE Hotline is to be contacted immediately:

SAE Hotline: 888-746-7231
SAE Fax: 888-746-3293

The initial report of an SAE should include the following minimum information: an identifiable subject; the investigational product; an identifiable reporting source; and an event or outcome that can be identified as serious. The investigator or designee must also inform the IRB, in compliance with GCP and local IRB reporting guidelines, of an SAE, whether or not considered study-related. The initial written report to the CRO and to the IRB must be as complete as possible.

Questions regarding safety issues and any SAEs may be directed to the Medical Monitor:

Helen Thackray, MD, FAAP
Vice President, Clinical Development
GlycoMimetics, Inc.

Office Phone: (240) 243-1230
Office Fax: (240) 599-7680
24-Hour Contact Number (cell phone): (240) 476-2348
E-mail: hthackray@glycomimetics.com

The Sponsor will be responsible for reporting SAEs to the Food and Drug Administration as required.

7.4 Dose Limiting Toxicities and Study Stopping Rules

Dose-limiting toxicity will be defined as TEAEs of severity Grade 3-5 deemed by the investigator to be related to GMI-1070 per the AE section of this protocol (Sections 7.1 Monitoring, and 7.2, Reporting). For the purpose of determining Dose Limiting Toxicity, related AEs shall include “probably related” and “definitely related” as determined by the investigator. AEs will be graded according to the WHO Common Toxicity Criteria, or by severity of AE if not included in the WHO Criteria, as described in Section 7.2. Thus, the study will be temporarily halted for further review of safety data according to the following guidelines for toxicities deemed to be related to GMI-1070:

- If at least 2 Grade 3 events are observed in 1 subject.
- If at least 3 Grade 3 events are observed within a given dose level.
- If a Grade 4 or Grade 5 event is observed.
- If at least 2 subjects experience a VOC within 24 hours of receiving GMI-1070. A VOC is defined as an episode requiring a visit to a medical facility resulting in medical treatment for pain.

In addition, the study may be halted for further review:

- If, for any reason involving safety, the investigator deems continuing the study inappropriate.
- If, for any reason, the Sponsor deems continuing the study inappropriate.

Dosing will be halted in the event of unacceptable AEs as described above. Further review of the study data will be undertaken in this situation, and the action to be taken with the study will be determined together by the investigator and Sponsor.

If the study is halted temporarily or terminated, a written statement fully documenting the reasons for study halt or termination will be provided to the IRB.

8 DATA COLLECTION AND DATA MONITORING

8.1 Data Quality Assurance

This study will be organized, performed, and reported in compliance with the CRO's Standard Operating Procedures, protocols and working practice documents, and the requirements of national and international GCP and ICH guidelines. Compliance will be achieved through a combination of study specific audits of the investigational site and of the CRO's systems for data handling, analysis, and reporting.

8.2 Case Report Form and Source Documentation

The investigator or appropriate designee will enter the information required by the protocol onto a CRF for each subject. A CRF manual will be provided to assist in correct CRF completion. Study participants must not be identified by name on any study documents. Upon enrollment, subjects will be assigned and identified by subject identification numbers.

Any data that are recorded on the CRFs without any prior written or electronic record must have the source clearly identified.

8.3 Data Management

Data from CRFs and other external data will be entered into a clinical database as specified in the CRO's data management plan. Quality control and data validation procedures will be applied to ensure the validity and accuracy of the clinical database.

Data from CRFs and other external data will be reviewed and checked for omissions, apparent errors, and values requiring further clarification using computerized and manual procedures. Data queries requiring clarification will be documented and returned to the investigational site for resolution. Only authorized personnel will make corrections to the clinical database, and all corrections will be documented in an audit trail.

8.4 Data Monitoring

After study initiation, the investigational site will be visited by the CRO on behalf of the Sponsor to review the CRFs and source documents for completeness, accuracy, protocol compliance, subject safety, and adherence to guidelines outlined in the Study Operations Manual. The CRO representative will highlight any discrepancies found between source documents and the completed CRFs and ensure that appropriate site personnel address the discrepancies. When a discrepancy results in corrected CRF data, the correction

will be initialed and dated on the CRF. Uniform procedures will be discussed at the site initiation.

9 STATISTICAL ANALYSIS

The statistical analyses described in this section will be performed as further outlined in the Statistical Analysis Plan (SAP), which will be finalized prior to completion of the clinical portion of the study and will be included in the Clinical Study Report for this protocol. The SAP will give a detailed description of the summaries and analyses (primary and secondary) that will be performed. Changes from analyses planned in the protocol will be documented in the SAP. If changes to principal features stated in the protocol are required, these will be documented in a protocol amendment.

All variables will be summarized at specified time points by dose level. Categorical variables will be summarized using counts (n) and percents (%) and will be presented in the form n (%). Continuous variables (eg, height, weight) will be summarized using the number of observations, mean, standard deviation and/or standard error, and minimum and maximum values.

9.1 Analysis Populations

The following subject subsets will be defined for this study.

- Safety population: the set of subjects who received at least 1 dose of investigational product and have any follow-up information. All safety analyses, including analyses of AEs, clinical laboratory results, vital signs, and physical examinations will be conducted on the Safety population.
- Efficacy population: the set of subjects who received at least 1 dose of investigational product and at least 1 IVM measurement. The primary and secondary efficacy analyses will be performed on subjects from this population.
- PK population: The set of subjects who receive at least 1 dose of investigational product and who have sufficient post-dose blood samples taken to estimate C_{max} and AUC.

9.2 Statistical Methods

9.2.1 Subject Disposition

The disposition of subjects at all scheduled visits will be determined on the basis of evaluations made at each visit. The disposition will be summarized by dose level.

9.2.2 Demographics and Baseline Characteristics

Demographic and baseline information will be summarized using mean, standard deviation, and minimum and maximum values for continuous variables and the number and percentages for categorical variables.

Demographics and subject characteristics obtained at the baseline evaluation will be summarized descriptively using the Safety population. Demographics by dose level will be summarized with the following information: age, genotype, race, ethnicity, and weight.

Due to the small sample size, no inferential tests will be performed to statistically compare the dose levels on demographics and baseline characteristics.

9.2.3 Efficacy

The primary efficacy analysis will be performed on the Efficacy population. For the final analysis, mean microvascular blood flow and change from baseline will be summarized by time point. Change from baseline microvascular blood flow will be modeled longitudinally using a mixed effects model with a random subject effect and baseline microvascular blood flow and treatment as fixed effects.

Secondary efficacy outcome measures will include biomarkers of adhesion, inflammation, and downstream selectin effect in the blood. All of these secondary measurements will be analyzed using a mixed model similar to the primary efficacy endpoint.

9.2.4 Safety Data

Safety endpoints will be summarized by cohort and descriptively analyzed for differences across cohorts using a Fisher’s Exact Test as the data allow. All AEs, including SAEs, will be coded using the MedDRA dictionary. A by-subject listing of all AEs will be provided. Summaries will be provided for each of the following types of AEs:

- Treatment-emergent AEs (TEAEs);
- Treatment-emergent SAEs;
- Treatment-emergent SAEs that were reported as “possibly”, “probably”, or “definitely” related to study drug;
- TEAEs that were reported as “possibly”, “probably”, or “definitely” related to study drug;
- TEAEs by severity;
- TEAEs that lead to discontinuation of the study;
- TEAEs resulting in death.

TEAEs will be defined as those AEs that begin on or after the date/time of study drug administration. Drug-related AEs will include those AEs that are reported by the investigator as possibly, probably, or definitely related to the study drug. With the exception of the TEAEs by severity summary, all summaries will present the number of AEs and the number and percent of subjects having an AE by system organ class and by specific AE preferred term. The TEAEs by severity summary will only report the number and percent of subjects. For all percentages, the subjects in the Safety population by dose level will comprise the denominator.

In addition to these summary tables, subjects with treatment-emergent SAEs and subjects who discontinued prematurely due to a TEAE will be presented in separate subject listings. The listings will provide all of the information reported for that AE and will include the length of time from study drug administration to the occurrence of the AE. If any deaths occur in the study, a similar listing of all TEAEs for subjects who died will be provided.

9.2.5 Pharmacokinetic Analysis

Due to the limited number of plasma concentrations, PK parameters will be estimated by fitting a compartmental model derived from richer datasets in healthy volunteers (Studies GMI-1070-101 and GMI-1070-102).

PK parameters to be estimated will include:

CL	Total plasma clearance
CL_{D2}	Clearance from the peripheral (non-central compartment)
V_1	Volume of the central compartment
V_2	Volume of the peripheral (non-central compartment)
C_{max}	Maximum observed drug concentration
t_{max}	Time of the maximum drug concentration (obtained without interpolation)
α	Apparent distribution rate constant
β	Apparent terminal elimination rate constant
$t_{1/2}$	Apparent elimination half-life, calculated as $\ln(2)/\beta$

Urine

PK parameters will be computed from the individual urine concentrations using a noncompartmental approach. Appropriate validated PK software (eg, WinNonlin Professional) will be used.

U_e	The amount excreted in urine during each collection interval (C_{urine}), calculated as C_{urine} x urine volume.
CL_r	Renal clearance, calculated as $CL_r = Ue/AUC_{0\text{-}t}$. CL_r will be calculated for each collection interval and for the entire 8-hour steady-state study period.
Cum_{Ue}	Cumulative amount excreted in urine over the urine collection period.
% Dose	The percentage of drug recovered in urine, calculated as (Cum_{Ue})/Dose x 100.

Analysis of Pharmacokinetics

The secondary endpoint is the PK of the multiple doses of IV GMI-1070 as given in this study.

PK parameters will be summarized by dose level using descriptive statistics (arithmetic means, standard deviations, coefficients of variation, sample size [N], minimum, maximum and median). Figures will be created to display mean and individual (observed and model-predicted) analyte concentration-time curves.

Additional analyses will be performed as deemed necessary upon review of the data.

9.2.6 Vital Signs and Laboratory Parameters

For vital signs and continuous laboratory parameters, the observed value and the change in the value from baseline at each collection point during the treatment phase will be presented. Values will be analyzed using a generalized linear mixed model over the longitudinal measurements. This model will allow comparisons of each secondary outcome over the treatment groups, controlling for time and baseline measurement as fixed effects and subject as a random effect.

For categorical laboratory parameters that are collected in terms of abnormal or normal or can be classified into positive or negative results, the number and percent of subjects having values in each category will be presented at each scheduled collection time. For those categorical laboratory parameters that are reported in several descriptive categories (eg, urinalysis color), the number and percent of subjects by each category will be presented. Graphs displaying population trends over time may be produced.

For both vital signs and laboratory parameters, clinically significant results will be listed with relevant subject information for assessment.

9.2.7 Physical Examinations

Significant changes in physical examination over the study visits will be listed by subject and summarized across the study population as appropriate.

9.2.8 Investigational Product Interim Analysis

An interim analysis will be performed by site. An interim analysis will be done after every fourth subject at each clinical site completes the study to determine whether to change the dose level for the next group of 4 at that site. These 4 subjects will be examined to determine whether the dose of GMI-1070 was safe and effective (see Section 6.2.5). PK data will also be analyzed at this time. This analysis will be repeated for each group of 4 subjects at the clinical site level.

9.3 Statistical Considerations

9.3.1 Missing Data

Subjects with missing outcome data will not be included in the analysis. Subjects who drop out of the study prior to obtaining endpoint data from hour 24 will be replaced by the next available subject (see Section 4.4).

9.3.2 Sample Size

This study represents Phase 1/2 in the development of this compound in sickle cell patients, and therefore does not require large numbers of subjects for rigorous statistical analysis typically reserved for later phases of development. Based on the prior human PK data with this compound, as described in the Investigator's Brochure, it is believed that at least 4 subjects per dose level, receiving 1 loading dose and 1 additional dose, will provide enough information about the dose-response curve to allow us to determine a smaller range of dose levels of GMI-1070 for future studies. In addition, the total sample of 20 subjects in this study will provide safety data in support of proceeding with Phase 2 studies in sickle cell patients experiencing VOC.

10 STUDY ADMINISTRATION

10.1 Direct Access to Source Data/Documents

The investigator or designee must maintain, at all times and in a secure manner, the original primary records (ie, source documents), regardless of media of each subject's data. Examples of source documents are laboratory reports, drug

inventory, study drug label records, the medical record, and CRFs that are used as the source.

The investigator and institution agree that the Sponsor, its representatives, the IRB, and representatives from worldwide regulatory agencies will have the right, both during and after the clinical trial, to inspect the facilities and review and inspect all study-related original source records, regardless of media.

10.2 Quality Assurance/Quality Control

Activities relevant to the quality of the study are monitored as deemed appropriate by the Quality Assurance Division of the CRO.

All clinical data will undergo a 100% quality control check prior to clinical database lock. The Sponsor will review data listings prior to this lock.

10.3 Data Handling and Record Keeping

All requested information must be entered on CRFs. If an item is not available or is not applicable, this fact should be indicated without leaving a blank space. All information should be completed in black ink and never in pencil or other colored ink. A correction should be made by striking through the incorrect entry with a single line and by entering the correct information adjacent to it. The correction must be initialed and dated by the person making the correction. Each set of completed CRFs must be reviewed, signed, and dated by the investigator.

The investigator agrees to retain study records as required by the study contract and applicable statutes. The investigator or designee agrees to contact the Sponsor before destroying any study documentation. Should the investigator leave the site at which the study was conducted, the CRO/Sponsor will be contacted regarding the disposition of document storage. All documents pertaining to the trial will be kept in accordance with the 1996 ICH guidelines for GCP. If an investigator withdraws from the study (e.g., relocation), the records will be transferred to a mutually agreed upon designee (ie, another investigator).

10.4 Ethical Considerations

10.4.1 Basic Principles

This research will be carried out in accordance with the US Code of Federal Regulations (CFR), 21 Parts 50, 56, and 312; and the International Conference on Harmonisation Good Clinical Practice, Consolidated Guideline, of April 1996 (ICH E6).

10.4.2 Institutional Review Board

An IRB will review this protocol and the associated informed consent document. The study will not start until the IRB has approved the protocol or a modification thereof and the informed consent document. The IRB must be constituted and operate in accordance with the principles and requirements described in 21 CFR Part 56.

10.4.3 Protocol Amendments

Any change to this protocol significantly affecting the safety of subjects, the scope of the investigation, or the scientific quality of the study, requires a written protocol amendment that must be approved by the Sponsor, the investigator, and the IRB before implementation.

Examples of amendments requiring such approval are:

- An increase in drug dosage or duration of exposure of subjects
- A significant change in the study design (eg, addition or deletion of a group or period)
- An increase in the number of invasive procedures to which subjects are exposed
- Addition or deletion of a test procedure for safety monitoring

These requirements for approval should in no way prevent any immediate action from being taken by the investigator or Sponsor in the interest of preserving the safety of all subjects included in the study. If an immediate change to the protocol is felt to be necessary by the investigator and is implemented by him/her for safety reasons, the Sponsor should be notified within 2 working days and the IRB should be informed within a reasonable timeframe.

Changes to the protocol affecting only administrative aspects of the study do not require formal protocol amendments or IRB approval; however, the IRB must be kept informed of such administrative changes. Examples of administrative changes not requiring formal protocol amendments and IRB approval include:

- Minor changes in wording (ie, typographical errors, deletion of double-entry words or sentences, adding words for clarification)
- Clarification/modification of procedure instructions
- Change/correction of addresses or phone numbers
- Addition of new clinical sites

10.5 Additional Study Sites

The study may be expanded to additional clinical sites, at the discretion of the Sponsor.

11 REFERENCES

1 National Human Genome Research Institute. Learning about sickle cell disease. Available at: www.genome.gov/10001219. Accessed April 27, 2007.

2 Davis H, Roscoe M, Moore J, Gergen PJ. Cost of hospitalizations associated with sickle cell disease in the US. *Public Health Rep*. 1997;112:40-43.

3 Stuart MJ, Nagel RL. Sickle-cell disease. *Lancet*. 2004;364:1343-1360.

4 Frenette PS, Atweh GF. Sickle cell disease: old discoveries, new concepts, and future promise. *Journal of Clinical Investigation*. 2007;117:850-858.

5 Dampier C, Setty BNY, Eggleston B, Brodecki D, O'Neal P, Stuart M. Vaso-occlusion in children with sickle cell disease -- Clinical Characteristics and Biologic Correlates. *Journal of Pediatric Hematology/Oncology*. 2004;26(12):785-790.

6 Platt OS, Thorington BD, Brambilla DJ, et al. Pain in sickle cell disease: rates and risk factors. *N Engl J Med*. 1991;325:11-16.

7 Steinberg MH. Management of sickle cell disease. *N Engl J Med*. 1999;340:1021-1030.

8 American Academy of Pediatrics, Section on Hematology/Oncology and Committee on Genetics. Health supervision for children with sickle cell disease. *Pediatrics*. 2002;109:526-535.

9 Ballas SK. Pain management of sickle cell disease. *Hematology/Oncology Clinical of North America*. 2005;19:785-802.

10 Platt OS, Brambilla DJ, Rosse WF, et al. Mortality in sickle cell disease -- Life expectancy and risk factors for early death. *N Engl J Med*. 1994;330(23):1639-1644.

11 Bunn HF. Pathogenesis and treatment of sickle cell disease. *N Engl J Med*. 1997;337:762-769.

12 Okpala I. Leukocyte adhesion and the pathophysiology of sickle cell disease. *Current Opinion in Hematology*. 2006;13:40-44.

13 Richeimer S. The Richeimer Pain Update, Richeimer Pain Medical Group. December 2000, http://www.helpforpain.com/arch2000dec.htm. Accessed 11 December 2008.

14 Blum A, Yeganeh S, Peleg A, et al. Endothelial function in patients with sickle cell anemia during and after sickle cell crises. *Journal of Thrombosis and Thrombolysis*. 2005;19(2):83-86.

15 Kato GJ, Martyr S, Blackwelder WC, et al. Levels of soluble endothelium-derived adhesion molecules in patients with sickle cell disease are associated with pulmonary

hypertension, organ dysfunction, and mortality. *British Journal of Haematology*. 2005;130:943-953.

16 Turhan A, Weiss LA, Mohandas N, Coller PS, Frenette PS. Primary role for adherent leukocytes in sickle cell vascular occlusion: A new paradigm. *Proc Natl Acad Sci*. 2002;99:3047-3051.

17 Kaul DK, Hebbel RP. Hypoxia/reoxygenation causes inflammatory response in transgenic sickle mice but not in normal mice. *The Journal of Clinical Investigation*. 2000;106(3):411-420.

18 Zennadi R, Moeller BJ, et al. Epinephrine-induced activation of LW-mediated sickle cell adhesion and vaso-occlusion in vivo. *Blood*. 2007;110(7):2708-2717.

19 Biberthaler P, Langer S. Comparison of the New OPS Imaging Technique with Intravital Microscopy: Analysis of the Colon Microcirculation. *Eur Surg Res*. 2002;34:124-128.

20 Gupta S, Akerman S, et al. Intravital microscopy on a closed cranial window in mice: a model to study trigeminovascular mechanisms involved in migraine. *Cephalalgia*. 2006;26(11):1294-1303.

21 Lindell L, Lani H, et al. Intravital microscopy of leukocyte-endothelial dynamics using the Heidelberg confocal laser microscope in scleritis and allergic conjunctivitis. *Molecular Vision*. 2006;12:1302-1305.

22 Devaraj S, Cheung AT, et al. Evidence of Increased Inflammation and Microcirculatory Abnormalities in Patients With Type 1 Diabetes and Their Role in Microvascular Complications. *Diabetes*. 2007;56:2790-2796.

23 Cheung ATW, Chen PCY, et al. Microvascular abnormalities in sickle cell disease: a computer-assisted intravital microscopy study. *Blood.* 2002;99: 3999-4005.

24 Cheung ATW, Harmatz P, et al. Correlation of abnormal intracranial vessel velocity, measured by transcranial Doppler ultrasonography, with abnormal conjunctival vessel velocity, measured by computer-assisted intravital microscopy, in sickle cell disease. *Blood*. 2001;97(11):3401-3404.

25 Cheung ATM, Chan S, et al. Effects of Poloxamer 188 Treatment on Sickle Cell Vaso-Occlusive Crisis: Computer-Assisted Intravital Microscopy Study. *Journal of Investigative Medicine*. 2004;52(6):402-406.

26 Dimond A, Styles L, Kuypers F. Decreased microvascular perfusion in children with sickle cell disease by orthogonal polarization spectral imaging. Pediatric Academic Societies Annual Meeting 2008, Presentation 3215.2.

27 National Institutes of Health; Division of Blood Diseases and Resources. The management of sickle cell disease. 4th ed. NIH Publication No. 02-2117. Bethesda, MD: US Department of Health and Human Services; 2002.

12 APPENDICES

APPENDIX 1 WHO COMMON TOXICITY CRITERIA

These criteria may be found at http://www.fda.gov/cder/cancer/toxicityframe.htm (accessed 24 March 2009).

**Hematology**					
**Toxicity**	**Grade 0**	**Grade 1**	**Grade 2**	**Grade 3**	**Grade 4**
WBC (x103/l)	4	3.0 - 3.9	2.0 - 2.9	1.0 - 1.9	< 1.0
Platelets (x103/l)	WNL	75.0 - normal	50.0 – 74.9	25.0 - 49.9	< 25.0
Hemoglobin (g/dL)	WNL	10.0 - normal	8.0 - 9.9	6.5 - 7.9	< 6.5
Granulocytes/ Bands (x103/l)	2	1.5 - 1.9	1.0 - 1.4	0.5 - 0.9	< 0.5
Lymphocytes ($x10^3$/l)	2	1.5 - 1.9	1.0 - 1.4	0.5 - 0.9	< 0.5
Hemorrhage	none	mild, no	gross, 1 - 2 units transfusion per episode	gross, 3 - 4 units transfusion per episode	massive, > 4 units transfusion per episode
**Coagulation**					
**Toxicity**	**Grade 0**	**Grade 1**	**Grade 2**	**Grade 3**	**Grade 4**
Fibrinogen	WNL	0.99 - 0.75 x N	0.74 - 0.50 x N	0.49 - 0.25 x N	< 0.25 x N
Prothrombin time (Quick)	WNL	1.01 - 1.25 x N	1.26 - 1.50 x N	1.51 - 2.00 x N	> 2.00 x N
Partial thromboplastin time	WNL	1.01 - 1.66 x N	1.67 - 2.33 x N	2.34 - 3.00 x N	> 3.00 x N
**Metabolic**					
**Toxicity**	**Grade 0**	**Grade 1**	**Grade 2**	**Grade 3**	**Grade 4**
Hyperglycemia (mg/dL)	< 116	116 - 160	161 - 250	251 - 500	> 500 or ketoacidosis
Hypoglycemia (mg/dL)	> 64	55 - 64	40 - 54	30 – 39	< 30
Amylase	WNL	< 1.5 x N	1.5 - 2.0 x N	2.1 - 5.0 N	> 5.0 x N
Hypercalcemia (mg/dL)	< 10.6	10.6 - 11.5	11.6 – 12.5	12.6 – 13.4	13.5
Hypocalcemia (mg/dL)	> 8.4	8.4 - 7.8	7.7 - 7.0	6.9 - 6.1	6
Hypomagnesemia (mg/dL)	> 1.4	1.4 - 1.2	1.1 - 0.9	0.8 - 0.6	0.5

Abbreviations: WNL=within normal limits.

WHO COMMON TOXICITY CRITERIA (cont)					
**Gastrointestinal**					
**Toxicity**	**Grade 0**	**Grade 1**	**Grade 2**	**Grade 3**	**Grade 4**
Nausea	none	able to eat reasonable intake	intake significantly decreased but can eat	no significant intake	—
Vomiting	none	1 episode in 24 hours	2 - 5 episodes in 24 hours	6 - 10 episodes in 24 hours	> 10 episodes in 24 hours or requiring parenteral support
Diarrhea	none	increase of 2 - 3 stools/day over pre-Rx	increase of 4 - 6 stools/day, or nocturnal stools, or moderate cramping	increase of 7 - 9 stools/day, or incontinence, or severe cramping	increase of > 10 stools/day or grossly bloody diarrhea, or need for parenteral support
Stomatitis	none	painless ulcers, erythema, or mild soreness	painful erythema, edema, or ulcers but can eat solids	painful erythema, edema, or ulcers and cannot eat solids	requires parenteral or enteral support for alimentation
**Liver**					
**Toxicity**	**Grade 0**	**Grade 1**	**Grade 2**	**Grade 3**	**Grade 4**
Bilirubin (N = 17 µmol/L)	WNL	-----	< 1.5 x N	1.5 - 3.0 x N	> 3.0 x N
Transaminase (SGOT, SGPT)	WNL	2.5 x N	2.6 – 5.0 x N	5.1 - 20.0 x N	> 20.0 x N
Alk Phos or 5 nucleotidase	WNL	< 2.5 x N	2.6 – 5.0 x N	5.1 - 20.0 x N	> 20.0 x N
Liver- clinical	No change from baseline	-----	-----	Precoma	hepatic coma
**Kidney, bladder**					
**Toxicity**	**Grade 0**	**Grade 1**	**Grade 2**	**Grade 3**	**Grade 4**
Creatinine	WNL	< 1.5 x N	1.5 – 3.0 x N	3.1 - 6.0 x N	> 6.0 x N
Proteinuria	No change	1 (+) or < 0.3 g% or 3 g/L	2 - 3 (+) or 0.3 - 1.0 g% or 3 - 10 g/L	4 (+) or > 1.0 g% or > 10g/L	nephrotic syndrome
Hematuria	Negative	microscopic only	gross, no clots no Rx needed	gross and clots bladder irrigation	requires transfusion or cystectomy

WHO COMMON TOXICITY CRITERIA (cont)

Weight gain/loss

**Toxicity**	**Grade 0**	**Grade 1**	**Grade 2**	**Grade 3**	**Grade 4**
Weight gain/ loss	< 5.0 %	5.0 – 9.9 %	10.0 – 19.9 %	20.00%	-----

Pulmonary

Toxicity	Grade 0	Grade 1	Grade 2	Grade 3	Grade 4
Pulmonary	none or no change	asymptomatic, with abnormality in PFTs	dyspnea on significant exertion	dyspnea at normal level of activity	dyspnea at rest

Cardiac

**Toxicity**	**Grade 0**	**Grade 1**	**Grade 2**	**Grade 3**	**Grade 4**
Cardiac arrhythmias	none	asymptomatic, transient, requiring no therapy	recurrent or persistent, no therapy required	requires treatment	requires monitoring; or hypotension, or ventricular tachycardia or fibrillation
Cardiac function	none	asymptomatic, decline of resting ejection fraction by less than 20 % of baseline value	asymptomatic, decline of resting ejection fraction by more than 20% of baseline value	mild CHF, responsive to therapy	severe or refractory CHF
Cardiac ischemia	none	non-specific T- wave flattening	asymptomatic, ST and T wave changes suggesting ischemia	angina without evidence of infarction	acute myocardial infarction
Cardiac- pericardial	none	asymptomatic effusion, no intervention required	pericarditis (rub, chest pain, ECG changes)	symptomatic effusion; drainage required	tamponade; drainage urgently required
Hypertension	none or no change	asymptomatic, transient increase by greater than 20 mm Hg (D) or to > 150 / 100 if previously WNL. No treatment required.	recurrent or persistent increase by greater than 20 mm HG (D) or to > 150 / 100 if previously WNL. No treatment required.	requires therapy	hypertensive crisis
Hypotension	none or no change	changes requiring no therapy (including transient orthostatic hypotension)	requires fluid replacement or other therapy but not hospitalization	requires therapy and hospitalization; resolves within 48 hours of stopping the agent	requires therapy and hospitalization for > 48 hours after stopping the agent

Abbreviations: PFT=pulmonary function test, CHF=congestive heart failure,.

WHO COMMON TOXICITY CRITERIA (cont)

Neurologic

**Toxicity**	**Grade 0**	**Grade 1**	**Grade 2**	**Grade 3**	**Grade 4**
Neuro: sensory	none or no change	mild paresthesias; loss of deep tendon reflexes	mild or moderate objective sensory loss moderate paresthesias	severe objective sensory loss or paresthesias that interfere with function	-----
Neuro: motor	none or no change	Subjective weakness; no objective findings	mild objective weakness without significant impairment of function	objective weakness with impairment of function	paralysis
Neuro: cortical	none	mild somnolence or agitation	moderate somnolence or agitation	severe somnolence, (>50 % waking hours), agitation, confusion, disorientation, or hallucinations	coma, seizures, toxic psychosis
Neuro: cerebellar	none	Slight incoordination, dysdiadochokinesia	intention tremor, dysmetria, slurred speech, nystagmus	locomotor ataxia	cerebellar necrosis
Neuro: mood	no change	mild anxiety or depression	moderate anxiety or depression	severe anxiety or depression	suicidal ideation
Neuro: headache	none	mild	moderate or severe but transient	unrelenting and severe	-----
Neuro: constipation	none or no change	mild	moderate	severe	ileus >96 hours
Neuro: hearing	none or no change	Asymptomatic, hearing loss on audiometry only	Tinnitus	hearing loss interfering with function but correctable with hearing aid	deafness not correctable
Neuro: vision	none or no change	-----	-----	symptomatic subtotal loss of vision	blindness

Pain

**Toxicity**	**Grade 0**	**Grade 1**	**Grade 2**	**Grade 3**	**Grade 4**
Pain	none	mild	moderate	severe	req. narcotics

WHO COMMON TOXICITY CRITERIA (cont)

Skin

Toxicity	Grade 0	Grade 1	Grade 2	Grade 3	Grade 4
Skin	none or no change	scattered macular or papular eruption or erythema that is asymptomatic	scattered macular or papular eruption or erythema with pruritus or other associated symptoms	generalized symptomatic macular, papular or vesicular eruption	exfoliative dermatitis or ulcerating dermatitis

Alopecia

Toxicity	Grade 0	Grade 1	Grade 2	Grade 3	Grade 4
Alopecia	no loss	mild hair loss	pronounced or total hair loss	-----	-----

Allergy

Toxicity	Grade 0	Grade 1	Grade 2	Grade 3	Grade 4
Allergy	none	transient rash, drug fever < 38°C (100.4°F)	urticaria, drug fever 38°C (100.4°F), mild bronchospasm	serum sickness, bronchospasm requiring parenteral medication	anaphylaxis

Local

Toxicity	Grade 0	Grade 1	Grade 2	Grade 3	Grade 4
Local	none	pain	pain and swelling with inflammation or phlebitis	ulceration	plastic surgery indicated

Fever of unknown origin

Toxicity	Grade 0	Grade 1	Grade 2	Grade 3	Grade 4
Fever of unknown origin	none	37.1° - 38.0° C 98.7° - 100.4° F	38.1° - 40.0°C 100.5° - 104°F	> 40.0°C > 104.0°F for less than 24 hours	> 40.0°C (>104°F) for more than 24 hours or accompanied by hypotension

Infection

Toxicity	Grade 0	Grade 1	Grade 2	Grade 3	Grade 4
Infection	none	mild	moderate	severe	life-threatening

**WHO COMMON TOXICITY CRITERIA (cont)**	
**Additional events**	
**Toxicity**	
Asthenia	Analogous to Karnofsky index (WHO grading)
Chills	Analogous to fever
Peripheral edema	analogous to weight gain
Anorexia	analogous to weight loss

APPENDIX 2 DOCUMENT HISTORY - SUMMARY OF PROTOCOL AMENDMENTS

SUMMARY OF AMENDMENT 1

The following changes have been incorporated into Protocol Version 1.1: Amendment 1, dated 4 May 2009.

Section	Revision
Title Page	***Third investigational site added:***<br>Marilyn J. Telen, MD<br>Division Chief, Division of Hematology<br>Duke Comprehensive Sickle Cell Center<br>Division of Hematology<br>Duke University Medical Center<br>Box 2615<br>Durham, NC 27710<br>919-684-5378<br>email: telen002@mc.duke.edu<br><br>***Data manager added:***<br>Nancy Darrow<br>Amarex Clinical Research<br>20201 Century Blvd, 4th floor<br>Germantown, MD 20874<br>240-454-2874
Title Page<br>Protocol Signature Page	Added date of Amendment 1, version 1.1 of protocol.
Emergency Contact List	***Name of Rho Project Manager replaced with Clinical Research Associate:***<br>Carmen Wilburg<br>7313 Castle Drive<br>Dublin, CA 94568<br>Telephone (925) 828-7564<br>Fax: (925) 828-9017<br>Email: cmwilburg@comcast.net
Synopsis	***Sites:***<br>• Number of sites increased from 2 to 3.

Section	Revision
Synopsis	***Methodology (added modification of dosing paragraph for later-enrolling site[s]):***<br>• Note: If enrollment at a site begins after other sites have enrolled in the study, the safety, PK, and blood flow information gained by early-enrolling site(s) may be used to modify dosing by later-enrolling site(s).<br><br>After Dose Level A has been administered to at least 4 subjects at one site, and this site’s investigator and the Medical Monitor have determined it appropriate to proceed with another dose level as described above, a new site joining the study may proceed with either of the confirmed dose levels (Dose Level A, or the subsequent chosen dose level) if agreed by the new site’s investigator and Medical Monitor.
	Study Evaluations:<br>• Deleted biomarker sampling at 30 ± 10 minutes, and 2 ± 0.25, 4 ± 1 hours to be consistent with study schedule.<br>• Added wording for clarification (in bold text): Urine sampling for PK will be done over a 6-hour period **starting** immediately **after completion of** the infusion of the first dose.
	Inclusion criteria added (in bold text):<br>• Enrollment limited to subjects with SCD**-SS or SCD-Sβ^0-thal.**
	Exclusion criteria:<br>• All subjects enrolled in a hypertransfusion program now excluded, instead of only subjects with Hb S < 50%.<br>• Subjects with transfusions within the past 60 days now excluded, instead of within past 30 days.<br>• Use of inhaled steroids no longer an exclusion criterion, only systemic steroids.
Study Schedule	***Table 1: Schedule of Assessments:***<br>• Screening period defined as within 30 days of initiation of treatment, previously undefined.<br>• Specified pregnancy test as either urine or serum, previously serum only.<br>• Specified 6-hour urine collection for pharmacokinetics (PK), previously unspecified.<br>• Added concomitant medications to Day 28 observations.<br>• Added statement that during the Day 28 follow up telephone call, women of child-bearing potential will be asked if they

Section	Revision
	have had a positive pregnancy test since taking place in the study.
3	***Study Design:***<br>• Added Note: If enrollment at a site begins after other sites have enrolled in the study, the safety , PK, and blood flow information gained by early-enrolling site(s) may be used to modify dosing by later-enrolling site(s). After Dose Level A has been administered to at least 4 subjects at one site, and this site's investigator and the Medical Monitor have determined it appropriate to proceed with another dose level as described above, a new site joining the study may proceed with either of the confirmed dose levels (Dose Level A, or the subsequent chosen dose level) if agreed by the new site's investigator and Medical Monitor.<br>• Deleted biomarker sampling at 30 ± 10 minutes, and 2 ± 0.25, 4 ± 1 hours to be consistent with study schedule.
4.2	***Inclusion criteria:***<br>• Subjects with SCD-SS or SCD-Sβ^0-thal.
4.3	***Exclusion criteria:***<br>• All subjects enrolled in a hypertransfusion program now excluded, instead of only subjects with Hb S < 50%.<br>• Subjects with transfusions within the past 60 days now excluded, instead of within past 30 days.<br>• Use of inhaled steroids no longer an exclusion criterion, only systemic steroids.
5.1	***GMI-1070 (added modification of dosing paragraph for later-enrolling site[s]):***<br>• Note: If enrollment at a site begins after other sites have enrolled in the study, the safety, PK, and blood flow information gained by early-enrolling site(s) may be used to modify dosing by later-enrolling site(s).<br><br>After Dose Level A has been administered to at least 4 subjects at one site, and this site's investigator and the Medical Monitor have determined it appropriate to proceed with another dose level as described above, a new site joining the study may proceed with either of the confirmed dose levels (Dose Level A, or the subsequent chosen dose level) if agreed by the new site's investigator and Medical Monitor..
6.1.1	***Screening Visit:***

Section	Revision
	• Screening period defined as within 30 days of initiation of treatment, previously undefined.
6.2.1.3	***Electrocardiogram:***<br>• Added requirement for a 12-lead ECG to study procedure section.
6.2.1.4	***AST (SGOT)***<br>• Added to list of clinical laboratory evaluations.<br>***Urinalysis:***<br>• Leukocytes changed to leukocyte esterase
6.2.1.5	***Pregnancy Test:***<br>• Specified pregnancy test as either urine or serum, previously serum only.
6.2.2	***Non-Safety Sampling:***<br>• Specified 6-hour urine collection for PK, previously unspecified.<br>• Added wording for clarification (in bold text): Urine sampling for PK will be done over a 6-hour period **starting at the beginning** of the first dose.<br>• Deleted biomarker sampling at 30 ± 10 minutes, and 2 ± 0.25, 4 ± 1 hours to be consistent with study schedule.
7.4	***Dose limiting toxicity:***<br>• Added Grade 5 to “If Grade 4 event is observed.”
Appendix 1	***WHO CTC:***<br>• Added web address and date accessed to link for http://www.fda.gov/cder/cancer/toxicityframe.htm.
Appendix 2	Added Summary of Amendment 1.

SUMMARY OF AMENDMENT 2

The following changes have been incorporated into Protocol Version 1.2: Amendment 2, dated 24 August 2009.

Change No.	Section(s)	Previous Wording	Revised Wording	Justification
1.	Emergency Contact List	Carmen Wilburg<br>7313 Castle Drive<br>Dublin, CA 94568<br>Telephone (925) 828-7564<br>Fax: (925) 828-9017<br>Email: cmwilburg@comcast.net	Carmen Wilb**e**rg<br>7313 Castle Drive<br>Dublin, CA 94568<br>Telephone (925) 828-7564<br>Fax: (925) 828-9017<br>Email: cmwilb**e**rg@comcast.net	Administrative correction to spelling of Ms. Wilberg's last name.
2.	Emergency Contact List	SAE Fax (888) 746-329	SAE Fax (888) 746-329**3**	Administrative correction - last number to fax information was missing
3.	Study Synopsis and Section 4.2 Inclusion Criteria	16. Age 18 to 45 years	17. Age 18 to **50** years	If medically appropriate for the study, subjects up to age 50 may be included.
4.	Study Synopsis and Section 4.3 Exclusion Criteria	16. Weight of ≥ 90 kg at screening	17. Weight of ≥ **100** kg at screening	If medically appropriate for the study, subjects up to 100 kg may be included.

<table>
<tr><th>Change No.</th><th>Section(s)</th><th>Previous Wording</th><th>Revised Wording</th><th>Justification</th></tr>
<tr><td>7.</td><td>Study Schedule</td><td>Table 1: Schedule of Assessments currently has
Hematology/Complete Blood Count at Screening, Baseline, Day 2, Day 3, Day 7</td><td>Table 1: Schedule of Assessments will add a Hematology/Complete Blood Count at Screening, Baseline, Hour 8 ± 2 hours, Day 2, Day 3 and Day 7.</td><td>Assess WBC level after dosing and prior to Day 2, to evaluate for a shorter term effect in WBC.</td></tr>
<tr><td>5.</td><td>Section 6.2.1.4 Clinical Laboratory Evaluations</td><td>Table 2: Clinical Laboratory Evaluations
<table><tr><th>Serum Chemistry</th><th></th></tr><tr><td>Blood urea nitrogen</td><td>ALT (SGPT)</td></tr><tr><td>Creatinine</td><td>AST (SGOT)</td></tr><tr><td>Electrolytes with glucose</td><td>Total bilirubin</td></tr><tr><td></td><td>Lactate dehydrogenase</td></tr></table></td><td>Table 2: Clinical Laboratory Evaluations
<table><tr><th>Serum Chemistry</th><th></th></tr><tr><td>Blood urea nitrogen</td><td>ALT (SGPT)</td></tr><tr><td>Creatinine</td><td>AST (SGOT)</td></tr><tr><td>Electrolytes with glucose</td><td>Total and Direct bilirubin</td></tr><tr><td></td><td>Lactate dehydrogenase</td></tr></table></td><td>Added direct bilirubin laboratory assessment to obtain a more comprehensive clinical understanding of hepatic effects of the study drug.</td></tr>
</table>

Change No.	Section(s)	Previous Wording	Revised Wording	Justification
6.	Section 7.2 Reporting	Second Paragraph: "Each sign or symptom reported will be graded in accordance with the WHO Common Toxicity Criteria (Appendix 2)"<br>Fourth Paragraph: "If the AE does not have a toxicity grade listed in Appendix 2…"	Second Paragraph: "Each sign or symptom reported will be graded in accordance with the WHO Common Toxicity Criteria (Appendix **1**)"<br>Fourth Paragraph: "If the AE does not have a toxicity grade listed in Appendix **1**…"	Corrected reference to incorrect appendix.

SUMMARY OF AMENDMENT 3

The following changes have been incorporated into Protocol Version 1.3 dated 27 October 2009.

Change No.	Section(s)	Previous Wording	Revised Wording	Justification
1	STUDY SYNOPSIS<br>Study Evaluations Section: Plasma Sampling for Biomarkers	• 8 ± 2, 24 ± 3, and 48 ±12 (PK only) hours after administration of the first dose of IV GMI-1070	• **4 ± 1,** 8 ± 2, 24 ± 3, and 48 ±12 (PK only) hours after administration of the first dose of IV GMI-1070	Revised to include plasma sampling at the 4 ± 1 hour timepoint for biomarkers for adhesion.
2	STUDY SCHEDULE<br>Table 1: Schedule of Assessments<br>Footnote #6	[6] Performed only at 8 hours.	[6] Performed only at **4 and** 8 hours.	Revised footnote to indicate that plasma sampling for biomarkers for adhesion will also be performed at the 4 hour timepoint.
3	3 STUDY DESIGN	• 8 ± 2, 24 ± 3, and 48 ±12 (PK only) hours after administration of the first dose of IV GMI-1070	• **4 ± 1,** 8 ± 2, 24 ± 3, and 48 ±12 (PK only) hours after administration of the first dose of IV GMI-1070	Revised to include plasma sampling at the 4 ± 1 hour timepoint for biomarkers for adhesion.
4	6.2.2 Non-Safety Sampling	• 8 ± 2, 24 ± 3, and 48 ±12 (PK only) hours after administration of the first dose of IV GMI-1070	• **4 ± 1,** 8 ± 2, 24 ± 3, and 48 ±12 (PK only) hours after administration of the first dose of IV GMI-1070	Revised to include plasma sampling at the 4 ± 1 hour timepoint for biomarkers for adhesion.
